# A novel fast vector method for genetic sequence comparison

**DOI:** 10.1038/s41598-017-12493-2

**Published:** 2017-09-22

**Authors:** Yongkun Li, Lily He, Rong Lucy He, Stephen S.-T. Yau

**Affiliations:** 1 Department of Mathematical Sciences, Tsinghua University, Beijing, 100084 PR China; 2 Department of Biological Sciences, Chicago State University, Chicago, IL60628 USA

## Abstract

With sharp increasing in biological sequences, the traditional sequence alignment methods become unsuitable and infeasible. It motivates a surge of fast alignment-free techniques for sequence analysis. Among these methods, many sorts of feature vector methods are established and applied to reconstruction of species phylogeny. The vectors basically consist of some typical numerical features for certain biological problems. The features may come from the primary sequences, secondary or three dimensional structures of macromolecules. In this study, we propose a novel numerical vector based on only primary sequences of organism to build their phylogeny. Three chemical and physical properties of primary sequences: purine, pyrimidine and keto are also incorporated to the vector. Using each property, we convert the nucleotide sequence into a new sequence consisting of only two kinds of letters. Therefore, three sequences are constructed according to the three properties. For each letter of each sequence we calculate the number of the letter, the average position of the letter and the variation of the position of the letter appearing in the sequence. Tested on several datasets related to mammals, viruses and bacteria, this new tool is fast in speed and accurate for inferring the phylogeny of organisms.

## Introduction

In the past few decades, a basic but crucial task in biology is comparing genes and proteins for predicting the structure and function of these biological sequences. Many methods have been proposed to compare genetic sequences. Traditionally, most of these approaches are the widely used alignment-based methods. In these methods, molecular sequences are optimally aligned based on selected scoring systems. The alignment-based methods often give high accuracy and may reveal the relationships among sequences. Some algorithms have been established and incorporated into softwares for sequence alignments^1,2^. However, one of the main drawback of these techniques is that they are very time-consuming and expensive in memory usage. As a result, alignment-free approaches have attracted more and more attention and have been applied to biological sequence comparison as well as phylogeny analysis recently^3–7^. The noticeable characteristic of these methods is to incorporate some *ad hoc* numerical feature of sequences. These methods are fast in computational speed when comparing genes and proteins. Generally, alignment-free are categorized into four groups: methods based on k-mer or word frequency, methods based on substrings, methods based on information theory, and methods based on graphical representation. Firstly, methods based k-mer or word frequency are very popular and studied extensively. The classic k-mer method was proposed to compare genetic sequences, in which it counts the frequencies of substrings with k letters appearing in respective sequences^8^. Afterwards, a lot of k-mer based methods have been developed and applied in sequence analysis and phylogeny for viruses and bacteria. Such as feature frequency profile (FFP)^9^ which counts the number of substrings with fixed length k occurring in a genome^9^ and a vector consisting of these numbers is formed for each genome, return time distribution (RTD)^10^, frequency chaos game representation (FCGR)^11^, an improved complete composition vector method (ICCV)^12^ which was proposed by optimizing composition vector (CV)^13^ and complete composition vector (CCV)^14^. ICCV is under the assumption of a uniform and independent model to estimate sequence information. Comparing with CCV and CV method, ICCV method is more robust and efficient in performing sequence comparison. Secondly, methods based on substrings employ the similarity of substrings in a pair of sequences. These algorithms are mostly used for string processing in computer science, like average common substring (ACS)^15^, k-mismatch average common substring approach (kmacs)^16^. Thirdly, methods based on information theory include global and local characterization of sequences and estimate genome entrop to regions. Among them base-base correlation (BBC)^17^, information correlation and partial information correlation (IC-PIC)^18^ are representative articles. Finally, methods based on graphical representation also play important roles in sequence comparison in alignment-free area^19–21^.

Although k-mer models are widely used in many biological studies, the position information and the properties of nucleotides is ignored in these methods. The second kind of methods are computationally expensive among alignment-free methods. For the third algorithms, most of them are alignment based. The graph based methods are often used to display some characteristics of sequences directly by curves. Therefore, it is better to use these information for sequences comparison. In this paper, we develop an 18 dimensional feature vector to characterize a DNA sequence. The vector includes the occurrence frequency of each of the four bases, the average position of nucleotides and the biochemical properties of nucleotides. To validate the advantages of our approach, we test it on several data sets and further make comparison with some state-of-the-art sequence analysis techniques. Our method performs as good as alignment methods in accuracy and spends far less time than them. For one viral dataset, it even provides better result than alignment methods. It also works better than k-mer based methods in accuracy.

## Results

To test the effectiveness of our new method, we employ it to different datasets, which include mammalian mitochondrial genomes, virus, and bacteria genomes. Some datasets are small sized and others are medium sized. The length of sequences ranges from ten thousands to several millions base pairs. For each dataset, using our method the multiple encoding vectors of its genetic sequences are calculated. To verify the efficiency of our algorithms, the FFP method is used to compare. We use MEGA to draw the phylogeny of datasets for the two methods. Unlike FFP which applies Jensen-Shannon Divergence to compute the pairwise distance between any two vectors, our new method use Euclidean distance. In the process of using FFP method, we choose k to be the minimum integer of *log*_4_(*n*), i.e. *k* = *floor*(*log*_4_(*n*)) (n is the minimum length of sequences studied). From the results we can see the efficiency of our new method is better than FFP on these datasets.

### Phylogeny of mammals

**Figure 1:**
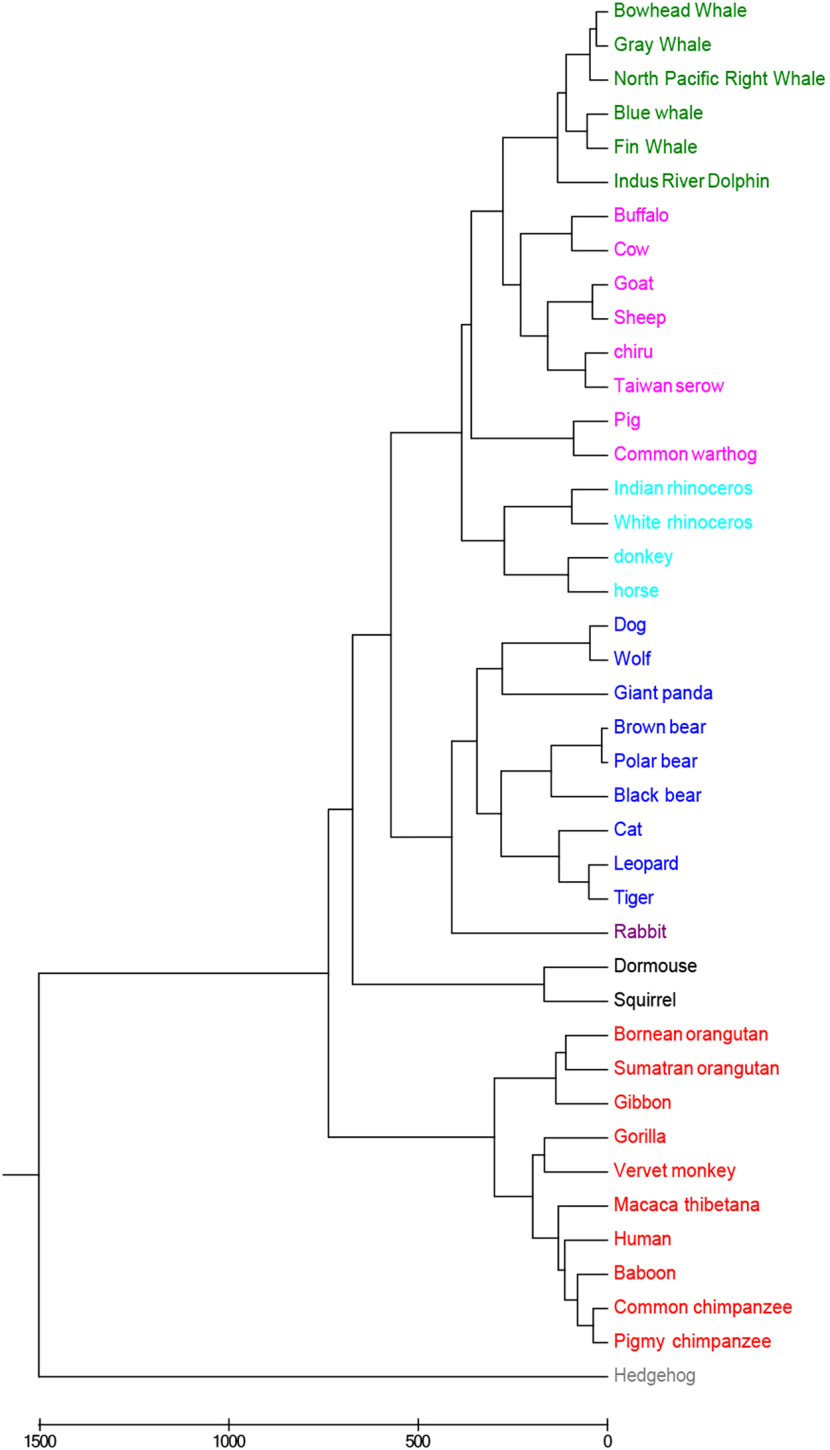
Phylogenetic tree of 41 mitochondrial genome sequences based on our multiple encoding vector method. The 41 species are correctly grouped into 8 clusters: *Primates* (red), *Cetacea* (green), *Artiodactyla* (pink), *Perissodactyla* (light green), *Rodentia* (black), *Lagomorpha* (dark red), *Carnivore* (blue), and *Erinaceomorpha* (grey).

The first benchmark dataset contains 41 mammalian complete mitochondrial genomes (mtDNA) with about 16500 base pairs. In these mammals, the structure of mtDNA is circular and double-stranded. One strand of each mtDNA has rich guanine referred to as the heavy strand and the other strand includes rich cytosine referred to as the light strand. In our study, the heavy strands of mtDNA are chosen. These sequences are not highly conserved and have a fast mutation rate^22^. Using our proposed method along with UPGMA algorithm, the phylogenetic tree of 41 mitochondrial genomes is constructed. As displayed in Fig. 1, the 41 species are correctly grouped into 8 clusters: *Primates* (red), *Cetacea* (green), *Artiodactyla* (pink), *Perissodactyla* (light green), *Rodentia* (black), *Lagomorpha* (dark red), *Carnivore* (blue), and *Erinaceomorpha* (grey). According to the FFP method, length of substrings is chosen to be 7 and thus the phylogeny of the 41 species is obtained. The phylogenetic tree is provided in the supplementary file. As displayed in Fig. S1, the 8 clusters are not classified well. The four species in *Perissodactyla* are improperly clustered into two clades. *Indus River Dolphin* from *Cetacea* is separated from other species from *Cetacea*. The *Carnivore*, *Primates* and *Artiodactyla* clades are all mistakenly divided into more than one group. Obviously, for this dataset our new method gets a greater result.

### Phylogeny of influenza A viruses

**Figure 2:**
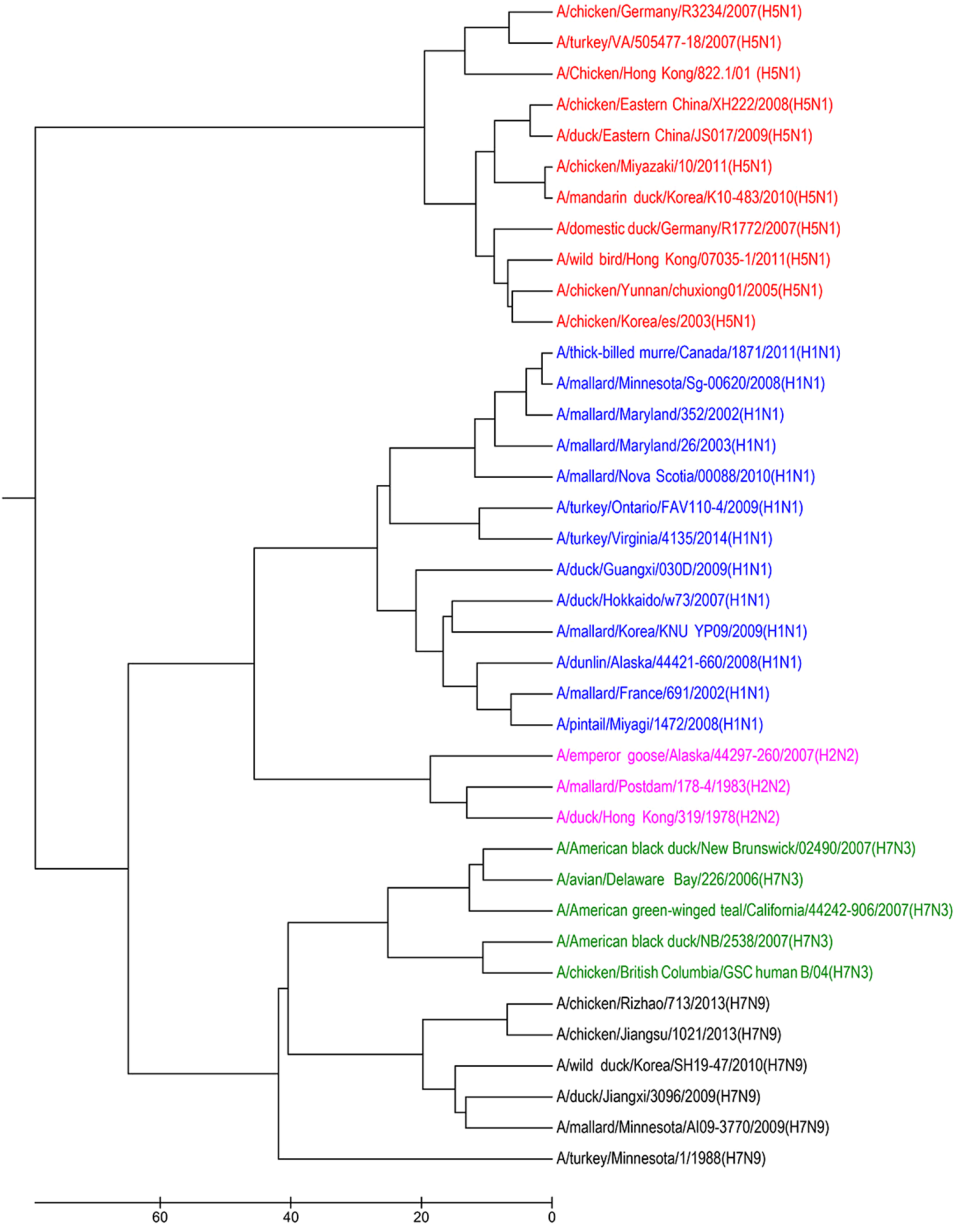
The UPGMA phylogenetic tree of 38 influenza A viruses based on our multiple encoding vector method.

Influenza A viruses may cause influenza disease in birds and mammals^23^. Influenza A viruses are single-stranded, negative-sense, segmented RNA viruses. The genome of flu virus contains around 13500 nucleotides and is divided into 8 segments. The subtypes of Influenza A viruses are classified according to an H number for the type of hemagglutinin (H) and an N number for the type of neuraminidase (N) in viral surfaces^24^. The viruses have 18 different H serotypes (H1-H18) and 11 different N (N1-N11) serotypes. For instance, the recently appearing H7N3 is an Influenza A strain with a type 7 hemagglutinin (H) and a type 3 neuraminidase (N). The several subtypes of Influenza A viruses are able to infect many species including wild birds, swines, dogs, horses, and even humans. It is known that H1N1, H1N2, and H3N2 subtypes can circulate among humans. In this study, we utilize Segment 6 gene encoding N (neuraminidase) of Influenza A virus to perform phylogenetic analysis. Using our method, the 38 flu viruses are correctly clustered into five groups as show in Fig. 2. Our result is consistent with previous study^25^. According to the FFP method, the choice for length of k-mer of these viruses is 5 and their phylogenetic tree is constructed. As shown in Fig. S2 (in the supplementary file), however, some of the H1N1 viruses and some H5N1 viruses are placed together. Therefor, on this dataset our method is also superior than FFP.

### Phylogeny of Human rhinovirus

**Figure 3:**
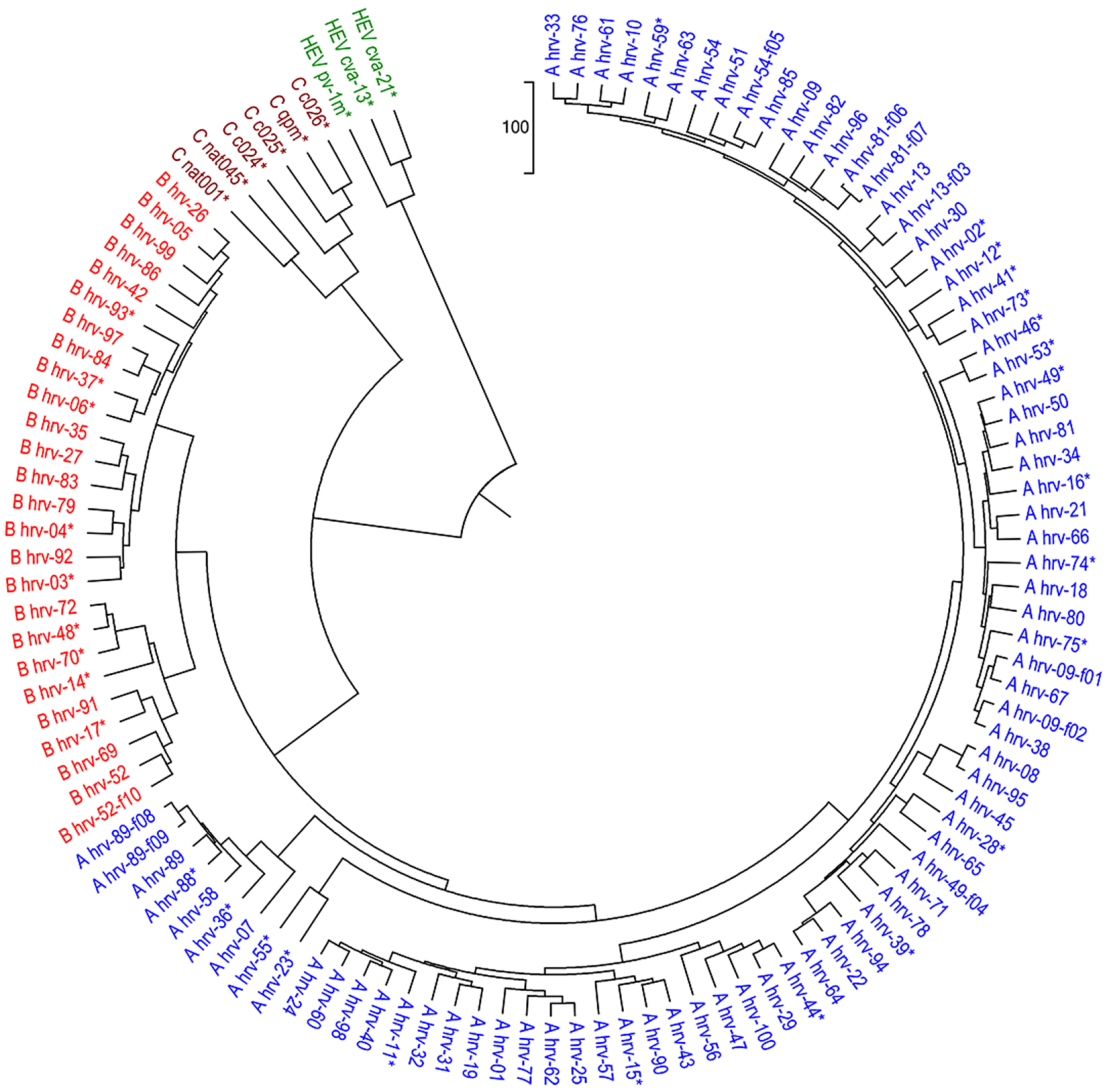
The UPGMA phylogenetic tree of 113 human rhinoviruses and 3 HEV-C viruses based on our multiple encoding vector method.

To test our proposed method, we perform the phylogeny of 116 Human rhinoviruses (HRVs). HRVs are the major cause of the common colds and lead to more than one-half of cold-like diseases. HRVs are single-stranded positive sense RNA viruses of about 7200 nucleotides in length. The genome of Human rhinovirus contains a single gene encoding one polyprotein, which is then cleaved to yield 11 proteins. HRVs belong to *Enterovirus* genus and *Picornaviridae* family. They form three phylogenetically distinct clusters: HRV-A, HRV-B, HRV-C^4,26^. In the previous work, 113 complete genomes composed of the three clusters are used to explore their evolutionary relationships. In addition, 3 HEV-C genomes were also added to the dataset as outgroup. Based on multiple sequence alignment (MSA), the evaluation was very time-consuming although the classification performed well. Unlike the MSA, our new approach is very fast to finish conversion from the genome sequences to 18 dimensional vectors. As shown in Fig. 3, the three groups of 116 genomes are identified and the remaining 3 outgroup viruses are clustered together alone. According to the FFP method, the length of words chosen for this dataset is 6 and the phylogenetic tree of 116 HRVs is obtained. As shown in Fig. S3 (in the supplementary file), two HRV-A viruses are mixed with the outlying HEV-C viruses.

### Phylogeny of ebolavirus

The *Ebolavirus* genus includes five species: Ebola virus (formerly Zaire ebolavirus, EBOV), Sudan virus (SUDV), Bundibugyo virus (BDBV), Tai Forest virus (TAFV), and Reston virus (RESTV)^27^. Ebola viruses are single-strand negative sense RNA viruses whose genomes consist of about 18900 bases. Each ebolavirus genome encodes seven proteins in which glycoprotein is the only viral protein on the surface of ebolavirus. The first case of human infected by EBOV was reported in 1976 in Zaire (currently the Democratic Republic of the Congo (DRC))^28^. Recently the epidemic starting in 2014 in Guinea is unprecedent because it is the first outbreak in West Africa and the magnitude is largest among known outbreaks.

**Figure 4:**
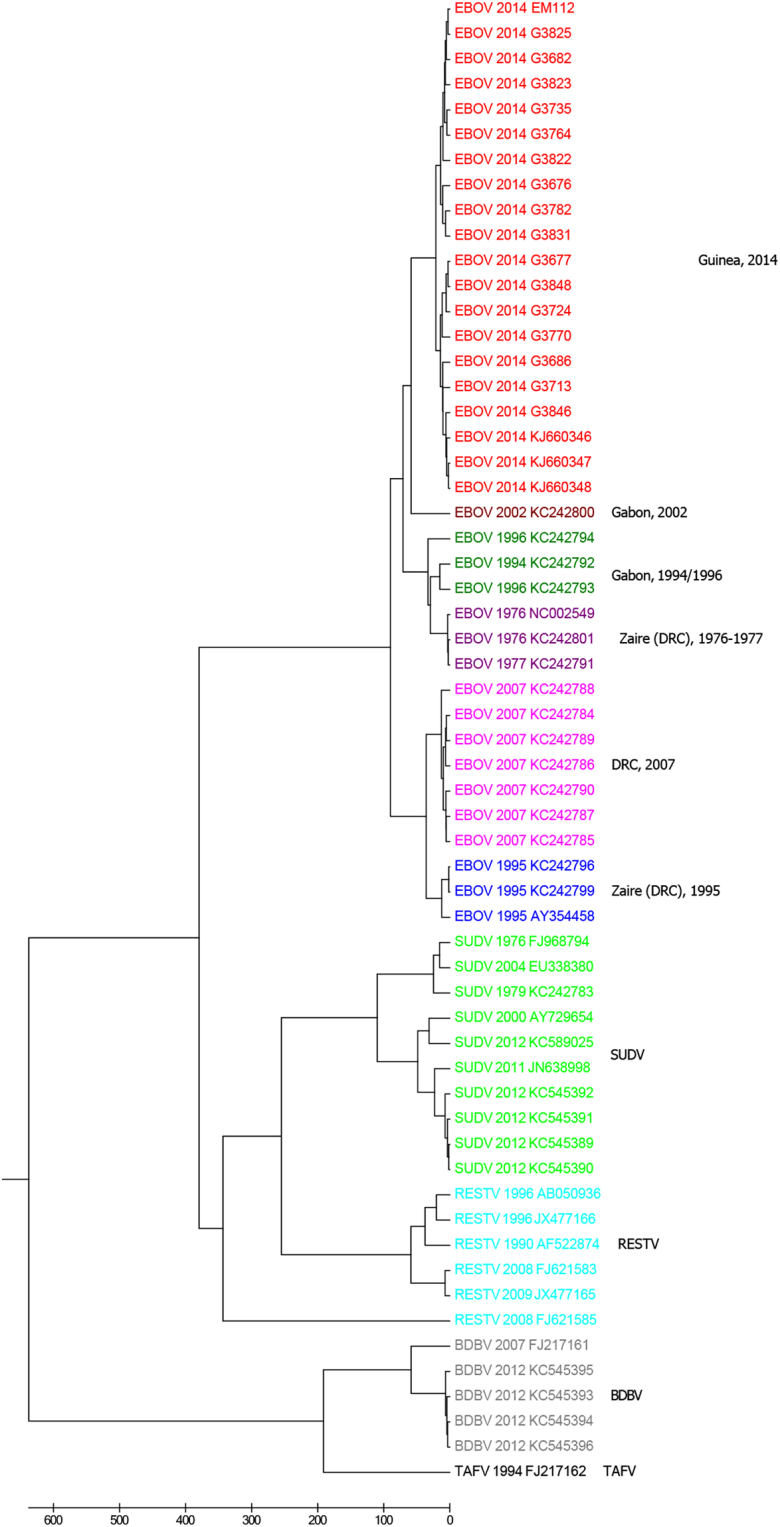
The UPGMA phylogenetic tree of 59 viruses in *Ebolavirus* genus based on our multiple encoding vector method. DRC = Democratic Republic of Congo, EBOV = Ebola virus, SUDV = Sudan virus, BDBV = Bundibugyo virus, TAFV = Tai Forest virus, RESTV = Reston virus.

To understand EBOV epidemics in humans and their relationships with other viruses in genus *Ebolavirus*, we apply the novel numerical vector method to construction of the phylogenetic tree of 59 viruses in *Ebolavirus* genus. As shown in Fig. 4, the five species are exactly separated. The EBOV strains from the confirmed pandemics form a lineage independent of the other four species in genus *Ebolavirus*. The EBOV strains in the 2014 epidemic in Guinea are clustered together. The EBOV strains in Zaire (DRC) pandemic in 1976–1977 are placed together as a clade. The three EBOV strains from the 1995 outbreak in Zaire (DRC) form a clade. The EBOV strains in DRC pandemic in 2007 are clustered together. The strains from the two epidemics occurred in Gabon are divided into two different clusters according to the outbreak periods. The SUDV branch is clustered with the RESTV branch. BDBV and TAFV viruses are positioned together. Our results are consistent with those in previous work^27,29^. Since the viral genomes in this dataset have about 19 kilobases, the k-mer length is thus 7. The phylogenetic relationships among these viruses using FFP method are depicted in Fig. S4 (in the supplementary file). However, the SUDV branch is not clustered with RESTV branch in this figure, which is not consistent with the confirmed taxonomy of *Ebolavirus* genus.

### Phylogeny of coronavirus

Coronaviruses are enveloped, single-stranded, positive-sense RNA viruses within the family *Coronaviridae*. Their genomes in length range from 25,000 to 32,000 nucleotides. Some subtypes of coronaviruses can also infect humans and cause many respiratory and gastrointestinal diseases with highly variable severity. Recently, the emergent Middle East respiratory syndrome coronavirus (MERS-CoV) had high case-fatality rate and caused outbreak of severe respiratory disease in Arabian Peninsula. As a result of pandemics from coronaviruses especially the SARS, the classification and evolutionary relationships among these viruses have been extensively investigated.

**Figure 5:**
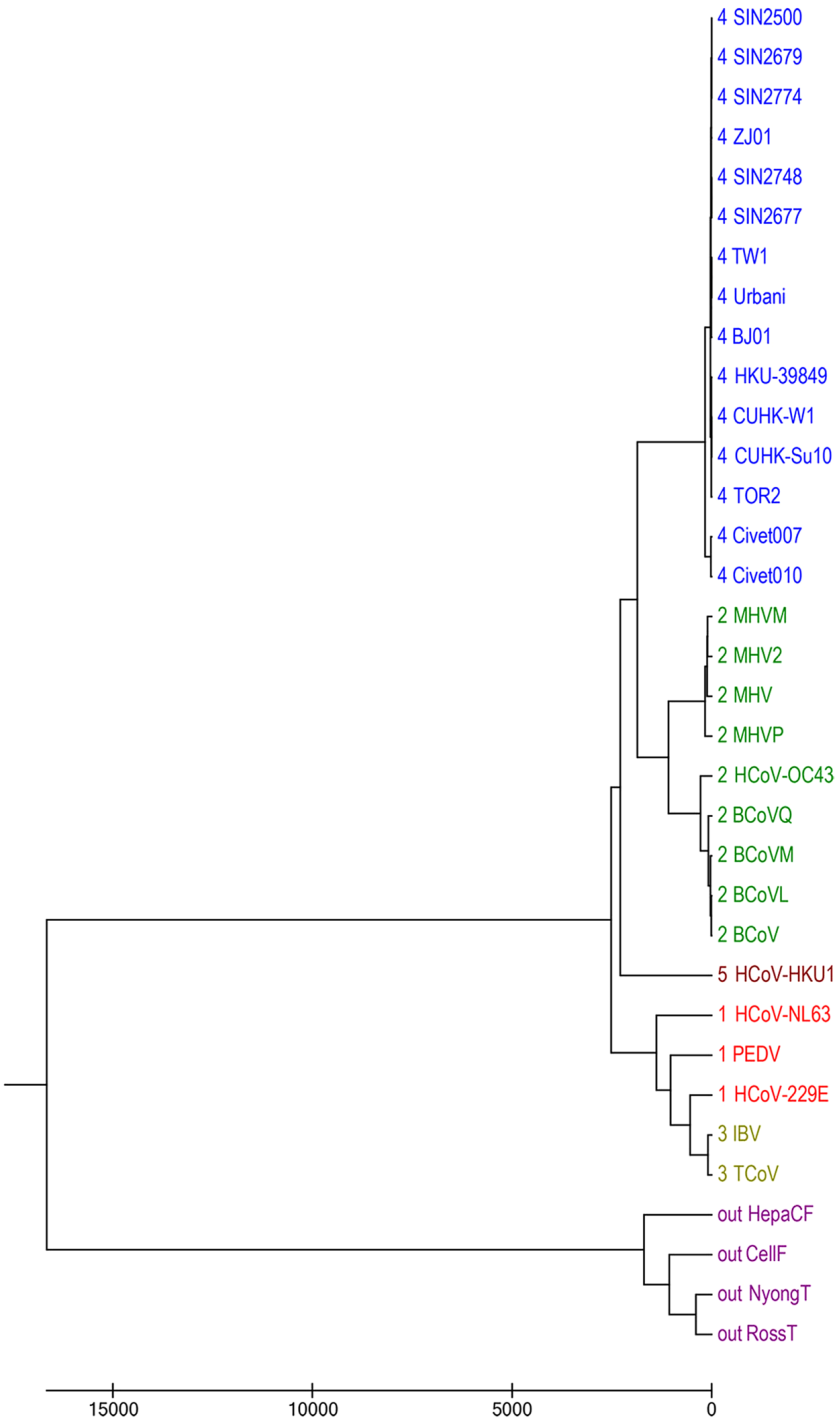
The UPGMA phylogenetic tree of 30 coronaviruses and 4 non-coronaviruses based on our multiple encoding vector method.

We apply our 18 dimensional vector method to a widely studied dataset containing 30 coronaviruses and 4 extra non-coronaviruses as outgroup. The newly discovered human coronavirus NL63 was clustered together with human coronavirus 229E (group 1)^30^. At the same time, it was positioned away from HCoV-OC43 (group 2) and SARS (group 4). The new isolated HCoV-HKU1 was thought as a distinct member of group 2^31^. However, the HCoV-HKU1 was designated as a member of a new group 5^32^. Using our novel method, the phylogeny of the coronaviruses is reconstructed based on the UPGMA algorithm. As shown in Fig. 5, HCoV-NL63, and HCoV-229E are grouped into group 1 as validated in the previous research^33^. Group 2, group 4, and new group 5 are separated in the tree, which is consistent with the studies in published papers^31,32^. According to the FFP method, the length of substrings chosen for these organisms is 6 and the phylogeny is constructed. As displayed in Fig. S5 (in the supplementary file) the HCoV-229E virus from group 1 is not correctly classified into group 2. Besides, the four viruses as outgroup are not clustered together. It means for this dataset our new method still has advantage over FFP.

### Phylogeny of bacteria

**Figure 6:**
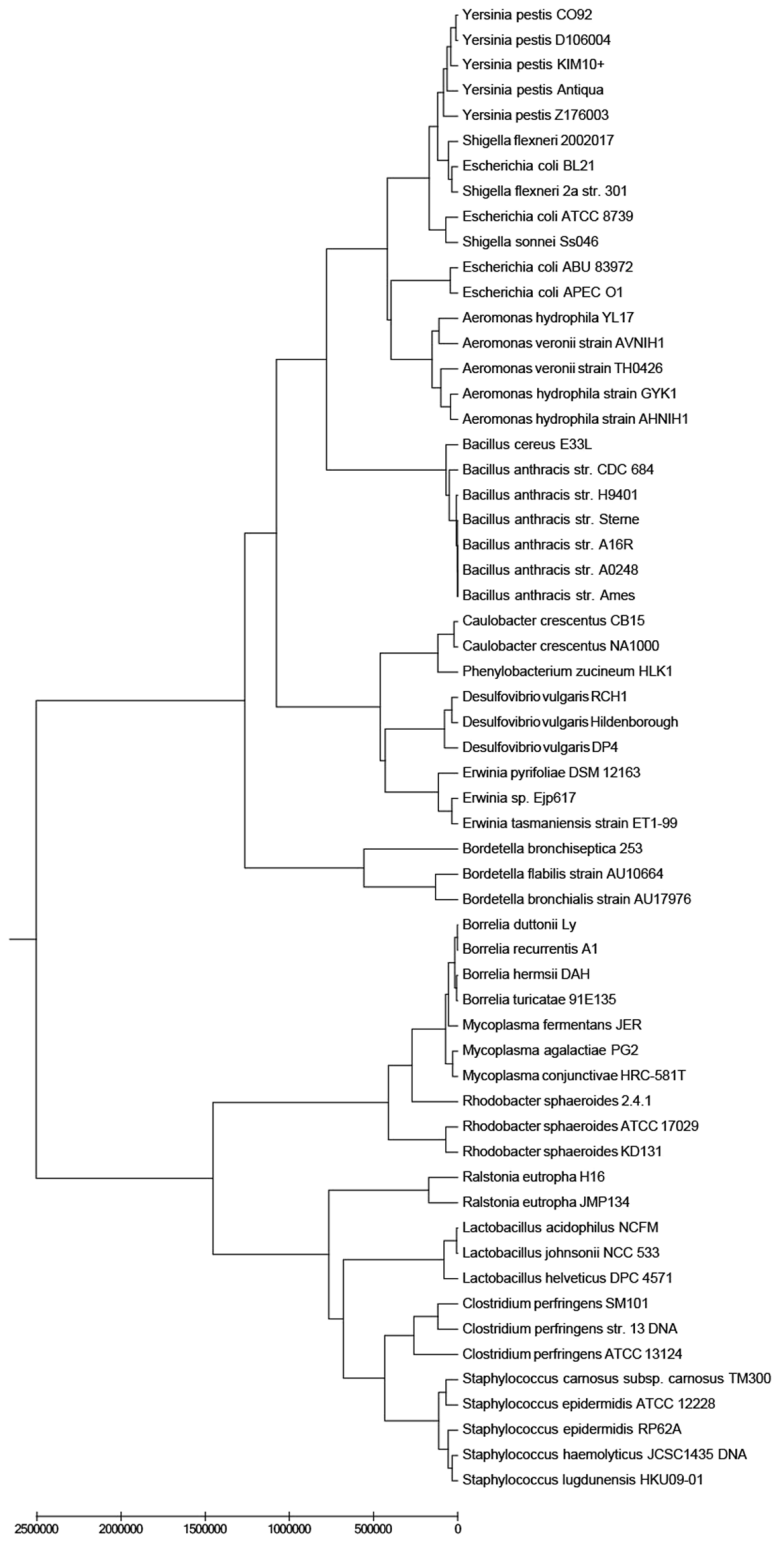
The UPGMA phylogenetic tree of 59 bacteria from 15 families based on our multiple encoding vector method.

Bacteria as one kind of prokaryotes are abundant on the earth. The phylogeny of these species has been an crucial topic in biology. As a result, the classification of bacteria based on whole genome sequences have attracted more and more attention. Due to long genomic sequences of bacteria with more than 1 million base pairs (Mb), the tradition multiple sequence alignment methods are computationally infeasible to perform phylogeny. To test our model, we analyze a dataset consisting of 59 bacterial species. This set contains 15 families: *Aeromonadaceae*, *Alcaligenaceae*, *Bacilleceae*, *Borreliaceae*, *Burkholderiaceae*, *Caulobacteraceae*, *Clostridiaceae*, *Desulfovibrionaceae*, *Enterobacteriaceae*, *Erwiniaceae*, *Lactobacillaceae*, *Mycoplasmataceae*, *Rhodobacteraceae*, *Staphylococcaceae*, *Yersiniaceae*. The genome length in this dataset mainly range from 3 to 10 Mb. As displayed in Fig. 6, the 15 families are well separated using our method. Based on the 9-mer FFP method, these families are also separated as illustrated in Fig. S6 (in the supplementary file). The two results are same for the phylogeny of 59 bacteria at the family level. However, our tree has some advantages at the phylum level. In Fig. 6, the three families *Lactobacillaceae*, *Clostridiaceae* and *Staphylococcaceae* from *phylum Bacilli* are clustered together. In the tree constructed by FFP model (Fig. S6), however, *Lactobacillaceae* are placed near to *Mycoplasmataceae* from phylum *Tenericutes*. Family *Desulfovibrionaceae* from phylum *Proteobacteria* are positioned in *phylum Bacilli*. Moreover, family *Clostridiaceae* from *phylum Bacilli* are grouped with *Borreliaceae* from *pylum Spirochaetes*. For the running time of algorithms, it is very time-consuming to calculate feature frequency profiles vectors with 4^9^ = 262144 dimensions. Since our vectors have 18 features, it takes only about 5 minutes to get these vectors.

## Discussion

Sequence comparison is crucial to understand the evolutionary relationships among organisms. Methods with alignment usually perform well if the genetic sequences are homologous. However, results obtained with alignment may be unreliable due to genetic recombination and high mutation rates. Besides, these approaches are time-consuming and thus are not suitable to align long sequences and large volume of data. According to the results mentioned above, our proposed alignment-free method shows its advantages on accuracy over alignment-based methods. Compared to a state-of-the-art method based on k-mer, the new technique still has the merit to construct correct evolutionary relationships. Our method is applicable to phylogeny construction of many kinds of species including mammals, viruses, and bacteria.

Additionally, we also compare our tool to ClustalW algorithm^2^ which is one of the most popular alignment methods. All programs are implemented on a laptop with 8 GB RAM, Intel (R) Core (TM) i7-4500U CPU, and 1.80 GHz and the running time unit is minute. As listed in Table 1, our approach is very fast in computational speed. For the large data of 59 bacteria, ClustalW even can not finish the alignment, while our method takes only 5.61 minutes to produce those numerical vectors. We also find the result for influenza A data with clustalW is unfavourable. As shown in Fig. S7 (in the supplementary file), A/turkey/VA/505477-18/2007 (H5N1) virus is clustered with H1N1 viruses. This may be caused by reassortment events occurring among virions. Other results by ClustalW are fine and put in the supplemental document.

**Table 1:** Running time for our multiple encoding vector and ClustalW (minutes).

	mtDNA	Influenza A	HRV	ebolavirus	coronavirus	bacteria
ClustalW	285	1.83	472	617	314	—
Our method	0.12	0.002	0.09	6.42	0.34	5.3

Our method is very fast and has the potential to construct phylogeny for whole genomes such large as mammalian. Note that mammalian genomes are often divided into several chromosomes. To compute the position of nucleotides, we only choose chromosome X of mammals to do phylogenetic analysis. Our dataset includes the species: chimpanzee (*Pan troglodytes*), human (*Homo sapiens*), monkey (*Macaca mulatta*), gorilla (*Gorilla gorilla*), dog (*Canis familiaris*), horse (*Equus caballus*), mouse (*Mus musculus*), opossum (*Monodelphis domesticus*), and platypus (*Ornithorhynchus anatinus*). The length of these X chromosomes ranges from about 6 to 147 Mb. The accession numbers are listed in the supporting file. Based on the new multiple encoding vector, the UPGMA phylogenetic tree of nine mammals is obtained. As shown in Fig. S8 in the supporting file, the primates are clustered together. Platypus and Opossum are placed in the base of tree.

In spite of the good performance in speed and accuracy of our approach, there are still some room for improvement. The method is sensitive to length of sequences analyzed. The sequences used for phylogenetic analysis should be approximately complete. For whole genome sequences studied, the use of partial genomes may produce incorrect evolutionary relationships. If we only use a gene or segment to infer phylogeny of species, the gene sequences or segments for all organism investigated should be intact. Thus we need to carefully prepare the sequences of organisms before constructing their phylogeny. Although the multiple sequence alignment does not have this requirement, gaps are inserted to sequences to make their length same automatically, which consumes much time.

In conclusion, we formulate a novel 18 dimensional vector method to compare biological sequences. The novelty of this method is that some important chemical and physical properties of sequences are incorporated into this method. Traditionally, the alignment-based method often produces phylogeny of species with high accuracy. However, these methods are time-consuming in speed and can not deal with large datasets and long sequences. As comparison, our approach is very fast and suitable for large sized biological sequences. Most importantly, the results tested on several different datasets show that this vector method can provide correct evolutionary relationships of different kinds of species.

## Methods

Let ${\mathcal L}$ be the set of 4 bases, i.e., ${\mathcal L} =\{A,C,G,T\}$ and *Q* = (*s*_1_, *s*_2_, ..., *s*_*n*_) be a DNA sequence of length *n*, that is, ${s}_{i}\in  {\mathcal L} ,i=\mathrm{1,2,}\cdots ,n\mathrm{.}$ The four bases are categorized into two groups according to three kinds of chemical and physical properties. The *A* and *G* bases are purine denoted by the letter *R*. The *C* and *T* bases are pyrimidine represented by *Y*. In another way of grouping, the *A* and *C* nucleotides are amino denoted by *M* and the *G* and *T* are keto denoted by *K*. According to the *H* bound, the *G* and *C* have strong *H* bond and they are represented by the letter *S*. The *A* and *T* bases contain weak *H* bond and they are represented by *W*. We define three numerical values for each of the letters *R*, *Y*, *M*, *K*, *S* and *W*. To characterize the distribution of *R* and *Y* in the sequence *Q*, we first replace the letters *A* and *G* by *R* and replace the *C* and *T* by *Y* in the sequence *Q*. Then the sequence only contains two kinds of letters: *R* and *Y*. For *R*, define *w*_*R*_(⋅):{*R*, *Y*} → {0, 1} such that *w*_*R*_(*s*_*i*_) = 1 if *s*_*i*_ = *R* and 0 otherwise. For *R*, we define the ${n}_{R},{\mu }_{R},{D}_{2}^{R}$ to describe the number of *R*, the average position of *R* and the variation of the position of *R* appearing in the sequence *Q*.

1. Let ${n}_{R}={\sum }_{i\mathrm{=1}}^{n}{w}_{R}({s}_{i})$ denote the number of letter *R* occurring in *Q*.
2. Let ${\mu }_{R}={\sum }_{i\mathrm{=1}}^{n}i\cdot \frac{{w}_{R}({s}_{i})}{{n}_{R}}$ be the mean position at which the letter *R* appears.
3. Let ${D}_{2}^{R}={\sum }_{i\mathrm{=1}}^{n}\frac{{(i-{\mu }_{R})}^{2}{w}_{R}({s}_{i})}{{n}_{R}n}$ be the scaled 2-nd moment of positions of letter *R*.

**Figure 7:**
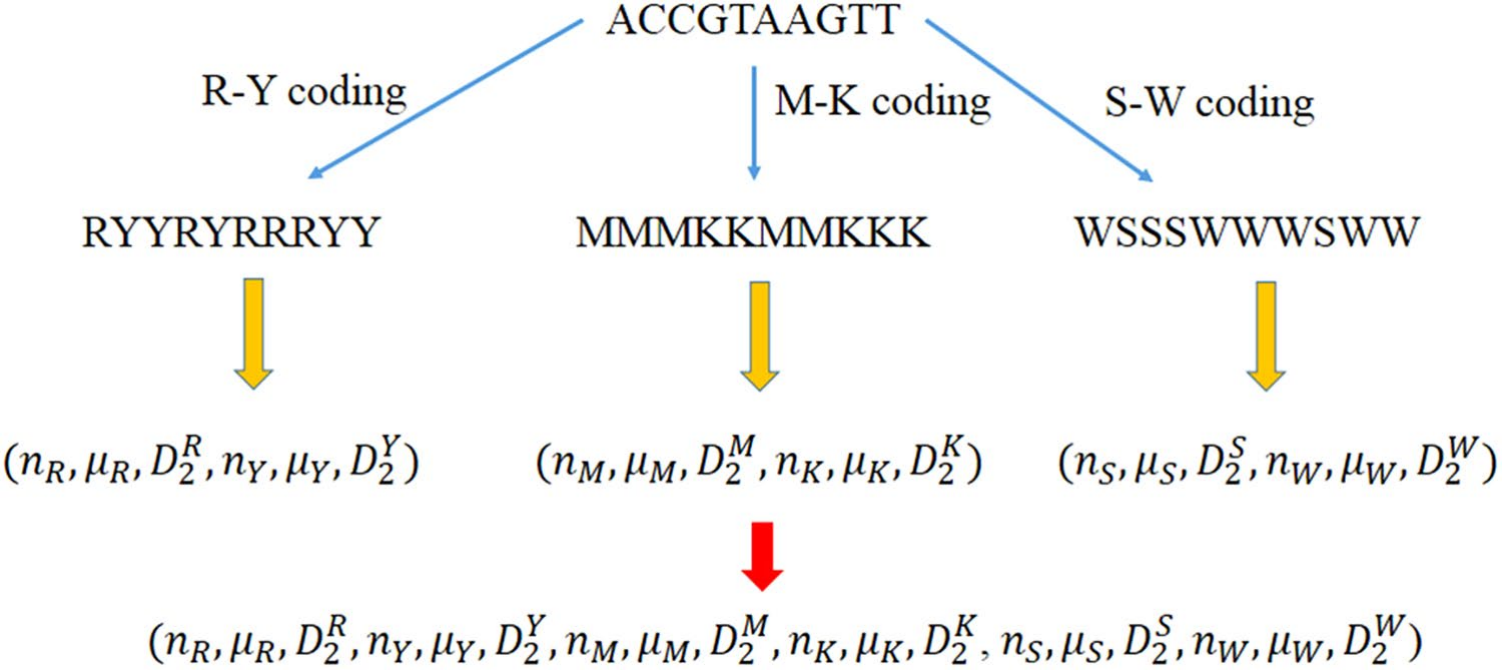
Flow chart of constructing 18 dimensional multiple encoding vector.

Similarly, for *Y* we define *w*_*Y*_(⋅):{*R*, *Y*} → {0, 1} such that *w*_*Y*_(*s*_*i*_) = 1 if *s*_*i*_ = *Y*, and 0 otherwise. Then we get three characteristics for *Y*: *n*_*Y*_, *μ*_*Y*_ and ${D}_{2}^{Y}$. For this kind of classification for nucleotides, six values are used to present the distribution of the four bases with respect to this chemical property. In the same way, we define other triplet for *M*, *K*, *S*, and *W*. Thus an 18 dimensional vector of the DNA sequence *Q* is defined by $({n}_{R},{\mu }_{R},{D}_{2}^{R},\cdots ,{n}_{W},{\mu }_{W},{D}_{2}^{W})\mathrm{.}$ We call this multiple encoding vector because it utilizes three groups of letters to encode the DNA sequence. The construction of this vector is shown in Fig. 7. Pairwise distance among vectors derived from the new method are computed using Euclidean distance. Then the distance matrix of all biological sequences is constructed. Based on the UPGMA algorithm, the phylogenetic tree of organisms is built using MEGA 6.0 software^37,38^.

### Data Availability

The datasets analysed during the current study are available in the “supplementary file” and the R source code in this paper is freely available to the public upon request.

## Electronic supplementary material

Supplementary PDF File

## References

[1] Altschul SF (1997). Gapped blast and psi-blast: a new generation of protein database search programs. Nucleic Acids Research.

[2] Larkin MA (2007). Clustal w and clustal x version 2.0. Bioinformatics.

[3] Vinga S, Almeida J (2003). Alignment-free sequence comparison-a review. Bioinformatics.

[4] Deng M, Yu C, Liang Q, He RL, Yau SS-T (2011). A novel method of characterizing genetic sequences: genome space with biological distance and applications. PLoS ONE.

[5] Yin C, Chen Y, Yau ST (2014). A measure of dna sequence similarity by fourier transform with applications on hierarchical clustering. Journal of Theoretical Biology.

[6] Li Y, Tian K, Yin C, He RL, Yau SS-T (2016). Virus classification in 60-dimensional protein space. Mol. Phylogenet. Evol..

[7] Hoang T, Yin C, Yau SS-T (2016). Numerical encoding of dna sequences by chaos game representation with application in similarity comparison. Genomics.

[8] Blaisdell BE (1989). Average values of a dissimilarity measure not requiring sequence alignment are twice the averages of conventional mismatch counts requiring sequence alignment for a computer-generated model system. Journal of Molecular Evolution.

[9] Sims GE, Jun S-R, Wu GA, Kim S-H (2009). Alignment-free genome comparison with feature frequency profiles (FFP) and optimal resolutions. Proc. Natl. Acad. Sci. USA.

[10] Kolekar P, Kale M, Kulkarni-Kale U (2012). Alignment-free distance measure based on return time distribution for sequence analysis: applications to clustering, molecular phylogeny and subtyping. Mol. Phylogenet. Evol..

[11] Hatje K, Kollmar M (2012). A phylogenetic analysis of the brassicales clade based on an alignment-free sequence comparison method. Front Plant Sci.

[12] Lu G, Zhang S, Fang X (2008). An improved string composition method for sequence comparison. BMC Bioinformatics.

[13] Gao L, Qi J (2007). Whole genome molecular phylogeny of large dsDNA viruses using composition vector method. BMC Evol. Biol..

[14] Wu X, Wan X-F, Xu D, Lin G (2006). Phylogenetic analysis using complete signature information of whole genomes and clustered Neighbour-Joining method. Int J Bioinform Res Appl.

[15] Ulitsky I, Burstein D, Tuller T, Chor B (2006). The average common substring approach to phylogenomic reconstruction. J. Comput. Biol..

[16] Leimeister C-A, Morgenstern B (2014). Kmacs: the k-mismatch average common substring approach to alignment-free sequence comparison. Bioinformatics.

[17] Cheng J, Zeng X, Ren G, Liu Z (2013). CGAP: a new comprehensive platform for the comparative analysis of chloroplast genome. BMC Bioinformatics.

[18] Gao Y, Luo L (2012). Genome-based phylogeny of dsDNA viruses by a novel alignment-free method. Gene.

[19] Jeffrey HJ (1990). Chaos game representation of gene structure. Nucleic Acids Research.

[20] Goldman N (1993). Nucleotide, dinucleotide and trinucleotide frequencies explain patterns observed in chaos game representations of DNA sequences. Nucleic Acids Research.

[21] Almeida JS, Carriço JA, Maretzek A, Noble PA, Fletcher M (2001). Analysis of genomic sequences by Chaos Game Representation. Bioinformatics.

[22] Brown WM, Prager EM, Wang A, Wilson AC (1982). Mitochondrial dna sequences of primates: Tempo and mode of evolution. J. Mol. Evol..

[23] Vijaykrishna D, Guan Y (2010). Reassortment of pandemic H1N1/2009 influenza A virus in swine. Science.

[24] Ghedin E (2005). Large-scale sequencing of human influenza reveals the dynamic nature of viral genome evolution. Nature.

[25] Yin C, Yau SS (2015). An improved model for whole genome phylogenetic analysis by fourier transform. J. Theor. Biol..

[26] Palmenberg AC, David Spiro RK (2009). Sequencing and analyses of all known human rhinovirus genomes reveal structure and evolution. Science.

[27] Gire SK (2014). Genomic surveillance elucidates ebola virus origin and transmission during the 2014 outbreak. Science.

[28] Holmes EC, Dudas G, Rambaut A, Andersen KG (2016). The evolution of ebola virus: Insights from the 2013–2016 epidemic. Nature.

[29] Baize S (2014). Emergence of zaire ebola virus disease in guinea. N. Engl. J. Med..

[30] Hoek LVD (2004). Identification of a new coronavirus. Nat. Med.

[31] Woo PCY (2005). Characterization and Complete Genome Sequence of a Novel Coronavirus, Coronavirus HKU1, from Patients with Pneumonia. J. Virol..

[32] Yu C, Qian L, Yin C, He RL, Yau ST (2010). A novel construction of genome space with biological geometry. DNA Res..

[33] Berkhout B (2004). Identification of a new human coronavirus. Nat. Med..

[34] Dai Q, Yang Y, Wang T (2008). Markov model plus k-word distributions: a synergy that produces novel statistical measures for sequence comparison. Bioinformatics.

[35] Wu GA, Jun S-R, Sims GE, Kim S-H (2009). Whole-proteome phylogeny of large dsdna virus families by an alignment-free method. Proc. Natl. Acad. Sci. USA.

[36] Xu Z, Hao B (2009). Cvtree update: a newly designed phylogenetic study platform using composition vectors and whole genomes. Nucleic Acids Research.

[37] Sokal RR (1958). A statistical method of evaluating systematic relationships. Univ. kansas Sci. bull.

[38] Tamura K, Stecher G, Peterson D, Filipski A, Kumar S (2013). Mega6: Molecular evolutionary genetics analysis version 6.0. Mol. Biol. Evol..

