# Supplementary material for: A novel fast vector method for genetic sequence comparison
Source: Sci Rep. 2017 Sep 22;7:12226. doi: 10.1038/s41598-017-12493-2 (PMC5610321; doi:10.1038/s41598-017-12493-2)

A novel fast vector method for genetic sequence comparison

Yongkun Li[1], Lily He[1], Rong Lucy He[2] and Stephen S.-T. Yau[1,*]

[1] Department of Mathematical Sciences, Tsinghua University, Beijing, 100084, PR China
[2] Department of Biological Sciences, Chicago State University, Chicago, Illinois, USA

* To whom correspondence should be addressed. Tel: +86 010 62787874; Email: yau@uic.edu

Table S1. Information of the 41 mammalian genomes

Accession Number	Abbreviation	Description	Genome Length (bp)
V00662	Human	Human	16569
D38116	Pig_Chim	Pigmy chimpanzee	16563
D38113	Com_Chim	Common chimpanzee	16554
D38114	Gorilla	Gorilla	16472
X99256	Gibbon	Gibbon	16521
Y18001	Baboon	Baboon	16389
AY863426	Ver_Monkey	Vervet monkey	16586
D38115	Bor_Oran	Bornean orangutan	16389
NC_002083	Sum_Oran	Sumatran orangutan	16499
U20753	Cat	Cat	16364
U96639	Dog	Dog	17009
AJ002189	Pig	Pig	16727
AF010406	Sheep	Sheep	16680
AF533441	Goat	Goat	16616
V00654	Cow	Cow	16640
AY488491	Buffalo	Buffalo	16338
EU442884	Wolf	Wolf	16355
EF551003	Tiger	Tiger	16774
EF551002	Leopard	Leopard	16990
X97336	Indian_Rhino	Indian Rhinoceros	16964
Y07726	White_Rhino	White Rhinoceros	16829
DQ402478	Black_Bear	Black Bear	16832
AF303110	Brown_Bear	Brown Bear	16868
AF303111	Polar_Bear	Polar Bear	17020
EF212882	Giant_Panda	Giant Panda	17017
AJ001588	Rabbit	Rabbit	16805
X88898	Hedgehog	Hedgehog	17245
NC_002764	Macaca_Thibet	Macaca Thibet	17447
AJ238588	Squirrel	Squirrel	16602
AJ001562	Dormouse	Dormouse	16507
X72204	Blue_Whale	Blue whale	16402
NC_005268	Bowhead Whale	Bowhead Whale	16390
NC_007441	chiru	chiru	16498
NC_008830	Common warthog	Common warthog	16719
NC_001788	donkey	donkey	16670
NC_001321	Fin Whale	Fin Whale	16398

NC_005270	Gray Whale	Gray Whale	16412
NC_001640	horse	horse	16660
NC_005275	Indus River Dolphin	Indus River Dolphin	16324
NC_006931	North Pacific Right Whale	North Pacific Right Whale	16386
NC_010640	Taiwan serow	Taiwan serow	16524

Table S2. Information of the 38 Influenza A viruses

**Accession Number**	**Description**	**Length (bp)**
HM370969	A/turkey/Ontario/FAV110-4/2009(H1N1)	1419
CY138562	A/mallard/Nova Scotia/00088/2010(H1N1)	1422
CY149630	A/thick-billed murre/Canada/1871/2011(H1N1)	1433
KC608160	A/duck/Guangxi/030D/2009(H1N1)	1398
AM157358	A/mallard/France/691/2002(H1N1)	1413
AB470663	A/duck/Hokkaido/w73/2007(H1N1)	1422
AB546159	A/pintail/Miyagi/1472/2008(H1N1)	1410
HQ897966	A/mallard/Korea/KNU YP09/2009(H1N1)	1410
EU026046	A/mallard/Maryland/352/2002(H1N1)	1433
FJ357114	A/mallard/Maryland/26/2003(H1N1)	1433
GQ411894	A/dunlin/Alaska/44421-660/2008(H1N1)	1413
CY140047	A/mallard/Minnesota/Sg-00620/2008(H1N1)	1433
KM244078	A/turkey/Virginia/4135/2014(H1N1)	1410
HQ185381	A/chicken/Eastern China/XH222/2008(H5N1)	1350
HQ185383	A/duck/Eastern China/JS017/2009(H5N1)	1350
EU635875	A/chicken/Yunnan/chuxiong01/2005(H5N1)	1350
FM177121	A/chicken/Germany/R3234/2007(H5N1)	1370
AM914017	A/domestic duck/Germany/R1772/2007(H5N1)	1350
KF572435	A/wild bird/Hong Kong/07035-1/2011(H5N1)	1350
AF509102	A/Chicken/Hong Kong/822.1/01 (H5N1)	1366
AB684161	A/chicken/Miyazaki/10/2011(H5N1)	1350
EF541464	A/chicken/Korea/es/2003(H5N1)	1350
JF699677	A/mandarin duck/Korea/K10-483/2010(H5N1)	1350
GU186511	A/turkey/VA/505477-18/2007(H5N1)	1370
EU500854	A/American black duck/NB/2538/2007(H7N3)	1453
CY129336	A/American black duck/New Brunswick/02490/2007(H7N3)	1428
CY076231	A/American green-winged teal/California/44242-906/2007(H7N3)	1420
CY039321	A/avian/Delaware Bay/226/2006(H7N3)	1434
AY646080	A/chicken/British Columbia/GSC_human_B/04(H7N3)	1453
KF259734	A/chicken/Rizhao/713/2013(H7N9)	1398
KF938945	A/chicken/Jiangsu/1021/2013(H7N9)	1404
KF259688	A/duck/Jiangxi/3096/2009(H7N9)	1413
KC609801	A/wild duck/Korea/SH19-47/2010(H7N9)	1426
CY014788	A/turkey/Minnesota/1/1988(H7N9)	1460
CY186004	A/mallard/Minnesota/AI09-3770/2009(H7N9)	1422
DQ017487	A/mallard/Postdam/178-4/1983(H2N2)	1467
CY005540	A/duck/Hong Kong/319/1978(H2N2)	1467
JX081142	A/emperor goose/Alaska/44297-260/2007(H2N2)	1457

Table S3. Information of 113 human rhinovirus genomes and three outgroup genomes

Accesion Number	Abbreviation	Length (bp)
AF499637	HEV_cva-13*	7458
AF546702	HEV_cva-21*	7406
AY751783	A_hrv-39*	7137
DQ473485	B_hrv-03*	7208
DQ473486	B_hrv-06*	7216
DQ473488	B_hrv-48*	7214
DQ473489	B_hrv-70*	7223
DQ473490	B_hrv-04*	7212
DQ473491	A_hrv-41*	7145
DQ473492	A_hrv-73*	7140
DQ473493	A_hrv-15*	7134
DQ473494	A_hrv-74*	7120
DQ473496	A_hrv-49*	7106
DQ473497	A_hrv-23*	7025
DQ473499	A_hrv-44*	7123
DQ473500	A_hrv-59*	7135
DQ473504	A_hrv-88*	7143
DQ473505	A_hrv-36*	7141
DQ473506	A_hrv-46*	7149
DQ473507	A_hrv-53*	7143
DQ473508	A_hrv-28*	7148
DQ473510	A_hrv-75*	7137
DQ473511	A_hrv-55*	7036
EF077279	C_nat001*	6944
EF077280	C_nat045*	7015
EF173414	A_hrv-11*	7125
EF173415	A_hrv-12*	7124
EF173420	B_hrv-17*	7219
EF173423	B_hrv-37*	7216
EF173425	B_hrv-93*	7215
EF186077	C_qpm*	7134
EF582385	C_c024*	7099
EF582386	C_c025*	7114
EF582387	C_c026*	7086
FJ445111	A_hrv-01	7137
FJ445112	B_hrv-05	7212
FJ445113	A_hrv-08	7108
FJ445114	A_hrv-09-f01	7134
FJ445115	A_hrv-09-f02	7133
FJ445116	A_hrv-13	7140
FJ445117	A_hrv-13-f03	7143
FJ445118	A_hrv-18	7119
FJ445119	A_hrv-19	7135
FJ445121	A_hrv-21	7134
FJ445122	A_hrv-22	7129
FJ445123	A_hrv-25	7126
FJ445124	B_hrv-26	7211
FJ445125	A_hrv-29	7123
FJ445126	A_hrv-31	7131
FJ445127	A_hrv-32	7133
FJ445128	A_hrv-33	7133
FJ445129	A_hrv-40	7138

FJ445130	B_hrv-42	7223
FJ445131	A_hrv-43	7129
FJ445132	A_hrv-45	7114
FJ445133	A_hrv-47	7132
FJ445134	A_hrv-49-f04	7109
FJ445135	A_hrv-50	7118
FJ445136	A_hrv-51	7152
FJ445137	B_hrv-52-f10	7216
FJ445138	A_hrv-54	7134
FJ445139	A_hrv-54-f05	7133
FJ445140	A_hrv-56	7136
FJ445141	A_hrv-57	7134
FJ445142	A_hrv-58	7140
FJ445143	A_hrv-60	7139
FJ445144	A_hrv-61	7139
FJ445145	A_hrv-62	7131
FJ445146	A_hrv-63	7141
FJ445147	A_hrv-65	7162
FJ445148	A_hrv-66	7139
FJ445149	A_hrv-67	7135
FJ445151	B_hrv-69	7211
FJ445152	A_hrv-71	7161
FJ445153	B_hrv-72	7216
FJ445154	A_hrv-77	7136
FJ445155	B_hrv-79	7224
FJ445156	A_hrv-80	7138
FJ445157	A_hrv-81	7116
FJ445158	A_hrv-81-f06	7116
FJ445159	A_hrv-81-f07	7116
FJ445160	A_hrv-82	7123
FJ445161	B_hrv-83	7230
FJ445162	B_hrv-84	7201
FJ445163	A_hrv-85	7140
FJ445164	B_hrv-86	7213
FJ445165	A_hrv-89-f09	7150
FJ445166	A_hrv-89-f08	7152
FJ445167	A_hrv-90	7124
FJ445168	B_hrv-91	7221
FJ445169	B_hrv-92	7233
FJ445170	A_hrv-95	7110
FJ445171	A_hrv-96	7134
FJ445172	B_hrv-97	7207
FJ445173	A_hrv-98	7133
FJ445174	B_hrv-99	7208
FJ445175	A_hrv-100	7140
FJ445176	A_hrv-07	7146
FJ445177	A_hrv-09	7132
FJ445178	A_hrv-10	7137
FJ445179	A_hrv-30	7093
FJ445180	A_hrv-38	7136
FJ445181	A_hrv-64	7129
FJ445182	A_hrv-76	7128
FJ445183	A_hrv-78	7145
FJ445184	A_hrv-89	7152
FJ445185	A_hrv-94	7132
FJ445186	B_hrv-27	7217
FJ445187	B_hrv-35	7224
FJ445188	B_hrv-52	7216

FJ445189	A_hrv-34	7119
FJ445190	A_hrv-24	7132
L05355	B_hrv-14*	7212
L24917	A_hrv-16*	7124
V01149	HEV_pv-1m*	7440
X02316	A_hrv-02*	7102

Table S4. Accesion number of 59 ebolaviruses

**Accession Number**	**Species**	**Genome Length (bp)**
FJ217161	BDBV_2007_FJ217161	18941
KC545393	BDBV_2012_KC545393	18940
KC545395	BDBV_2012_KC545395	18939
KC545394	BDBV_2012_KC545394	18938
KC545396	BDBV_2012_KC545396	18937
FJ217162	TAFV_1994_FJ217162	18936
AF522874	RESTV_1990_AF522874	18935
AB050936	RESTV_1996_AB050936	18934
JX477166	RESTV_1996_JX477166	18933
FJ621585	RESTV_2008_FJ621585	18932
FJ621583	RESTV_2008_FJ621583	18931
JX477165	RESTV_2009_JX477165	18930
FJ968794	SUDV_1976_FJ968794	18929
KC242783	SUDV_1979_KC242783	18928
EU338380	SUDV_2004_EU338380	18927
AY729654	SUDV_2000_AY729654	18926
JN638998	SUDV_2011_JN638998	18925
KC545389	SUDV_2012_KC545389	18924
KC545390	SUDV_2012_KC545390	18923
KC545391	SUDV_2012_KC545391	18922
KC545392	SUDV_2012_KC545392	18921
KC589025	SUDV_2012_KC589025	18920
KC242801	EBOV_1976_KC242801	18919

NC_002549	EBOV_1976_NC002549	18918
KC242791	EBOV_1977_KC242791	18917
KC242792	EBOV_1994_KC242792	18916
KC242793	EBOV_1996_KC242793	18915
KC242794	EBOV_1996_KC242794	18914
AY354458	EBOV_1995_AY354458	18913
KC242796	EBOV_1995_KC242796	18912
KC242799	EBOV_1995_KC242799	18911
KC242784	EBOV_2007_KC242784	18910
KC242786	EBOV_2007_KC242786	18909
KC242787	EBOV_2007_KC242787	18908
KC242789	EBOV_2007_KC242789	18907
KC242785	EBOV_2007_KC242785	18906
KC242790	EBOV_2007_KC242790	18905
KC242788	EBOV_2007_KC242788	18904
KC242800	EBOV_2002_KC242800	18903
KM034555	EBOV_2014_G3676	18902
KM034562	EBOV_2014_G3686	18901
KM233039	EBOV_2014_EM112	18900
KM034557	EBOV_2014_G3677	18899
KM034560	EBOV_2014_G3682	18898
KM233050	EBOV_2014_G3713	18897
KM233053	EBOV_2014_G3724	18896
KM233057	EBOV_2014_G3735	18895
KM233063	EBOV_2014_G3764	18894
KM233072	EBOV_2014_G3782	18893
KM233110	EBOV_2014_G3848	18892
KM233070	EBOV_2014_G3770	18891
KM233099	EBOV_2014_G3825	18890

KM233097	EBOV_2014_G3823	18889
KM233109	EBOV_2014_G3846	18888
KM233096	EBOV_2014_G3822	18887
KM233103	EBOV_2014_G3831	18886
KJ660346	EBOV_2014_KJ660346	18885
KJ660347	EBOV_2014_KJ660347	18884
KJ660348	EBOV_2014_KJ660348	18883

Table S5. Information of the 30 coronavirus genomes and the four outgroup genomes

**Accession Number**	**Abbreviation**	**Description**	**Length (bp)**
AF304460	1_HCoV-229E	Human coronavirus 229E, Group 1	27317
AF353511	1_PEDV	Porcine epidemic diarrhea virus strain, Group 1	28033
NC_005831	1_HCoV-NL63	Human coronavirus NL63, Group 1	27553
AY391777	2_HCoV-OC43	Human coronavirus OC43, Group 2	30738
U00735	2_BCoVM	Bovine coronavirus strain Mebus, Group 2	31032
AF391542	2_BCoVL	Bovine coronavirus isolate BCoV-LUN, Group 2	31028
AF220295	2_BCoVQ	Bovine coronavirus strain Quebec, Group 2	31100
NC_003045	2_BCoV	Bovine coronavirus, Group 2	31028
AF208067	2_MHVM	Murine hepatitis virus strain ML-10, Group 2	31233
AF201929	2_MHV2	Murine hepatitis virus strain 2, Group 2	31276
AF208066	2_MHVP	Murine hepatitis virus strain Penn 97-1, Group 2	31112
NC_001846	2_MHV	Murine hepatitis virus, Group 2	31357
NC_001451	3_IBV	Avian infectious bronchitis virus, Group 3	27608
EU095850	3_TCoV	Turkey coronavirus isolate MG10, Group 3	27657
AY278488	4_BJ01	SARS coronavirus BJ01, Group 4	29725
AY278741	4_Urbani	SARS coronavirus Urbani, Group 4	29727
AY278491	4_HKU-39849	SARS coronavirus HKU-39849, Group 4	29742
AY278554	4_CUHK-W1	SARS coronavirus CUHK-W1, Group 4	29736
AY282752	4_CUHK-Su10	SARS coronavirus CUHK-Su10, Group 4	29736
AY283794	4_SIN2500	SARS coronavirus isolate SIN2500, Group 4	29711
AY283795	4_SIN2677	SARS coronavirus isolate SIN2677, Group 4	29705
AY283796	4_SIN2679	SARS coronavirus isolate SIN2679, Group 4	29711
AY283797	4_SIN2748	SARS coronavirus isolate SIN2748, Group 4	29706
AY283798	4_SIN2774	SARS coronavirus isolate SIN2774, Group 4	29711
AY291451	4_TW1	SARS coronavirus TW1, Group 4	29729
NC_004718	4_TOR2	SARS coronavirus TOR2, Group 4	29751
AY297028	4_ZJ01	SARS coronavirus ZJ01, Group 4	29715
AY572034	4_Civet007	SARS coronavirus civet007, Group 4	29540
AY572035	4_Civet010	SARS coronavirus civet010, Group 4	29518
NC_006577	5_HCoV-HKU1	Human coronavirus HKU1, Group 5	29926
NC_001564	out_CellF	Cell fusing agent virus, *Flaviviridae* outgroup	10695
NC_004102	out_HepaCF	Hepatitis C virus, *Flaviviridae* outgroup	9646
NC_001512	out_NyongT	O'nyong-nyong virus, *Togaviridae* outgroup	11835
NC_001544	out_RossT	Ross River virus, *Togaviridae* outgroup	11657

Table S6. Information of the 59 bacterial genomes

Family	Species	Accession number	Genome Length(bp)
Aeromonadaceae	Aeromonas hydrophila strain AHNIH1	NZ_CP016380.1	9172850
	Aeromonas hydrophila strain GYK1	NZ_CP016392.1	9093803
	Aeromonas hydrophila YL17	CP007518.2	8856662
	Aeromonas veronii strain AVNIH1	NZ_CP014774.1	9014756
	Aeromonas veronii strain TH0426	NZ_CP012504.1	8935709
Bacillaceae	Bacillus anthracis str. A0248	CP001598.1	4983359
	Bacillus anthracis str. A16R	CP001974.1	8066192
	Bacillus anthracis str. Ames	AE016879.1	5062406
	Bacillus anthracis str. CDC 684	CP001215.1	5141453
	Bacillus anthracis str. H9401	NC_017729.1	8145239
	Bacillus anthracis str. Sterne	AE017225.1	5220500
	Bacillus cereus E33L	CP000001.1	8224286
Alcaligenaceae	Bordetella bronchialis strain AU17976	NZ_CP016171.1	9330944
	Bordetella bronchiseptica 253	NC_019382.1	8777615
	Bordetella flabilis strain AU10664	NZ_CP016172.1	9251897
Borreliaceae	Borrelia duttonii Ly	CP000976.1	4904312
	Borrelia hermsii DAH	CP000048.1	5299547
	Borrelia recurrentis A1	CP000993.1	5378594
	Borrelia turicatae 91E135	CP000049.1	5457641
Caulobacteraceae	Phenylobacterium zucineum HLK1	CP000747.1	8619521
	Caulobacter crescentus CB15	NC_002696.2	8540474
	Caulobacter crescentus NA1000	NC_011916.1	8698568
Clostridiaceae	Clostridium perfringens ATCC 13124	CP000246.1	5536688
	Clostridium perfringens SM101	CP000312.1	5615735
	Clostridium perfringens str. 13 DNA	BA000016.3	5694782
Desulfovibrionaceae	Desulfovibrio vulgaris DP4	CP000527.1	5773829
	Desulfovibrio vulgaris Hildenborough	AE017285.1	5931923

	Desulfovibrio vulgaris RCH1	CP002297.1	5852876
Erwiniaceae	Erwinia pyrifoliae DSM 12163	FN392235.1	6010970
	Erwinia sp. Ejp617	CP002124.1	6090017
	Erwinia tasmaniensis strain ET1-99	CU468135.1	6169064
Lactobacillaceae	Lactobacillus acidophilus NCFM	NC_006814.3	8303333
	Lactobacillus helveticus DPC 4571	NC_010080.1	8382380
	Lactobacillus johnsonii NCC 533	NC_005362.1	8461427
Mycoplasmataceae	Mycoplasma agalactiae PG2	CU179680.1	6485252
	Mycoplasma conjunctivae HRC-581T	FM864216.2	6564299
	Mycoplasma fermentans JER	CP001995.1	6643346
Burkholderiaceae	Ralstonia eutropha H16	AM260480.1	6722393
	Ralstonia eutropha JMP134	CP000091.1	6801440
Rhodobacteraceae	Rhodobacter sphaeroides 2.4.1	CP000144.2	6880487
	Rhodobacter sphaeroides ATCC 17029	CP000578.1	6959534
	Rhodobacter sphaeroides KD131	CP001151.1	7038581
Staphylococcaceae	Staphylococcus carnosus subsp. carnosus TM300	AM295250.1	7275722
	Staphylococcus epidermidis ATCC 12228	AE015929.1	7354769
	Staphylococcus epidermidis RP62A	CP000029.1	7433816
	Staphylococcus haemolyticus JCSC1435 DNA	AP006716.1	7512863
	Staphylococcus lugdunensis HKU09-01	CP001837.1	7591910
Yersiniaceae	Yersinia pestis Antiqua	CP000308.1	7670957
	Yersinia pestis CO92	AL590842.1	7750004
	Yersinia pestis D106004	CP001585.1	7829051
	Yersinia pestis KIM10+	AE009952.1	7908098
	Yersinia pestis Z176003	CP001593.1	7987145
Enterobacteriaceae	Escherichia coli ABU 83972	CP001671.1	6327158
	Escherichia coli APEC O1	CP000468.1	6406205
	Escherichia coli ATCC 8739	CP000946.1	4746218
	Escherichia coli BL21	CP001665.1	6248111

	Shigella flexneri 2002017	CP001383.1	7117628
	Shigella flexneri 2a str. 301	AE005674.2	7196675
	Shigella sonnei Ss046	CP000038.1	4825265

Table S7. Information of the 9 mammalian X chromosomes

Name	Assembly accession	Length (Mb)
Chimpanzee X chromosome	GCF_000001515.6	135.9
Human X chromosome	GCF_000001405.25	151.1
Monkey X chromosome	GCF_000772875.2	145.7
Gorilla X chromosome	GCF_000151905.1	143.0
Dog X chromosome	GCF_000002285.3	123.2
Horse X chromosome	GCF_000002305.2	121.6
Mouse X chromosome	GCF_000001635.25	163.5
Opossum X chromosome	GCF_000002295.2	73.0
Platypus X2 chromosome	GCF_000002275.2	5.5

Phylogenetic trees with the feature frequency profiles method

Figure S1. Phylogenetic tree of 41 mitochondrial genome sequences based on feature frequency profiles method using 7 mer. The 8 clusters are Primates (red), Cetacea (green), Artiodactyla (pink), Perissodactyla (light green), Rodentia (black), Lagomorpha (dark red), Carnivore (blue), and Erinaceomorpha (grey).

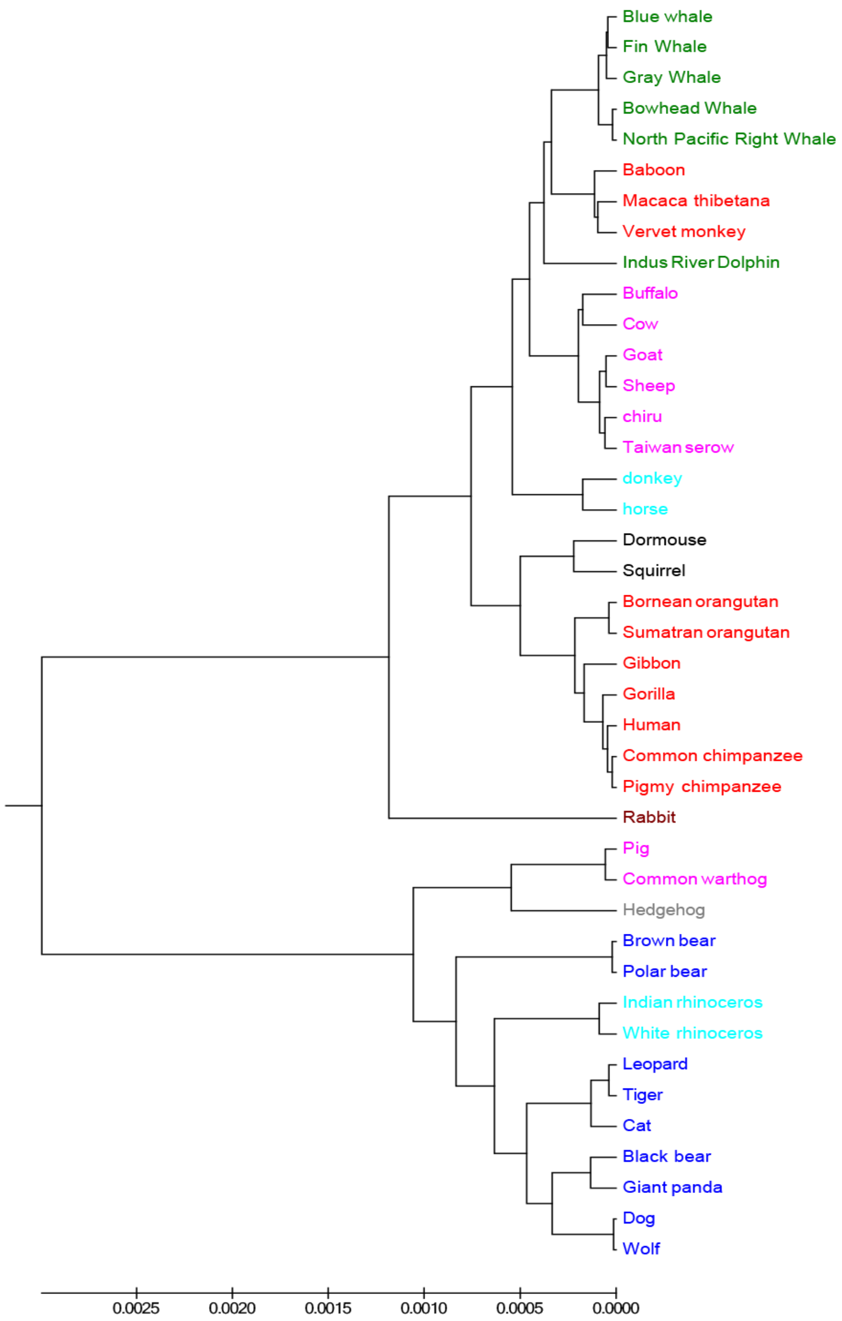

Figure S2. The UPGMA phylogenetic tree of 38 influenza A viruses based on feature frequency profiles method using 5 mer.

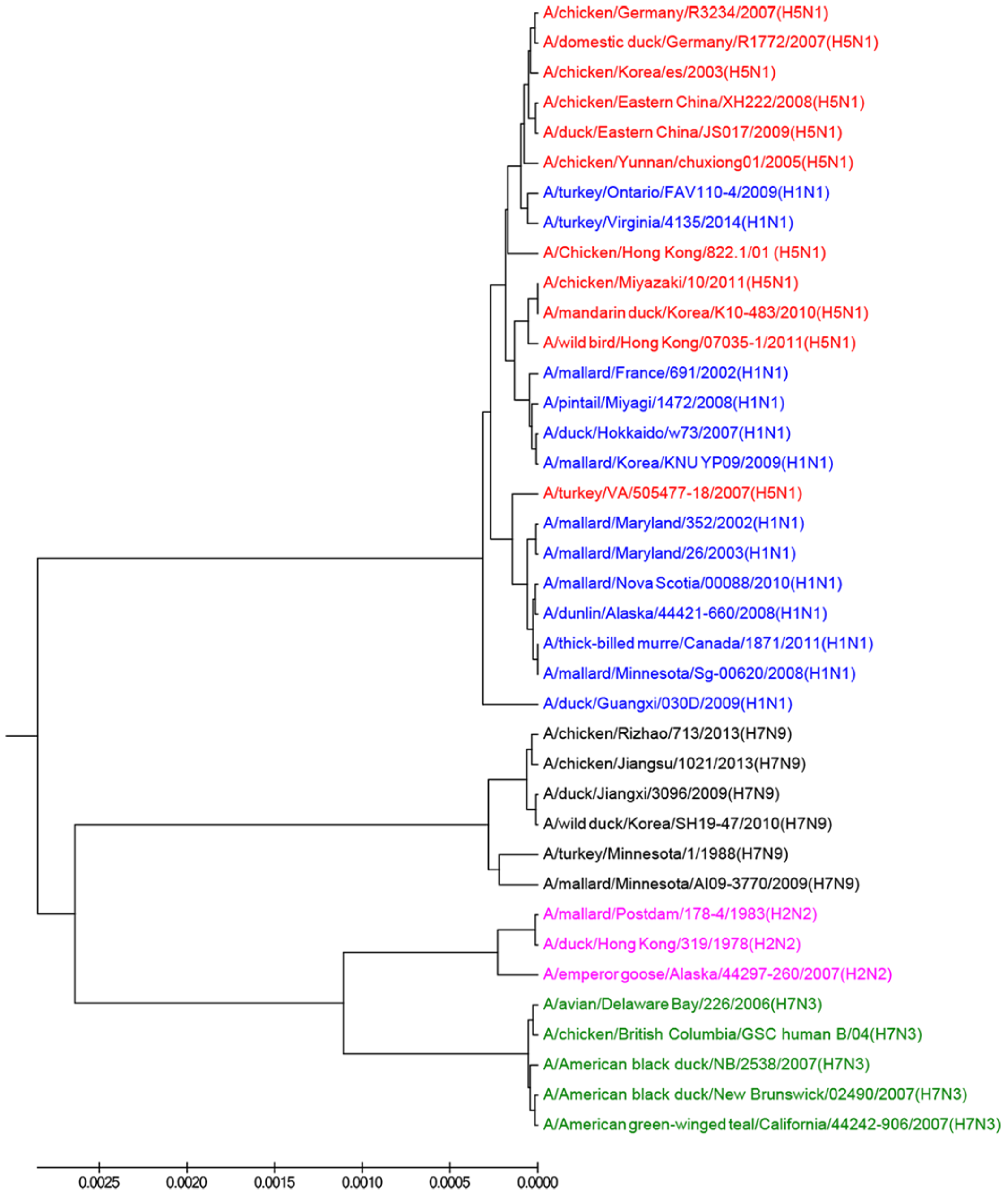

Figure S3. The UPGMA phylogenetic tree of human rhinoviruses based on feature frequency profiles method using 6 mer.

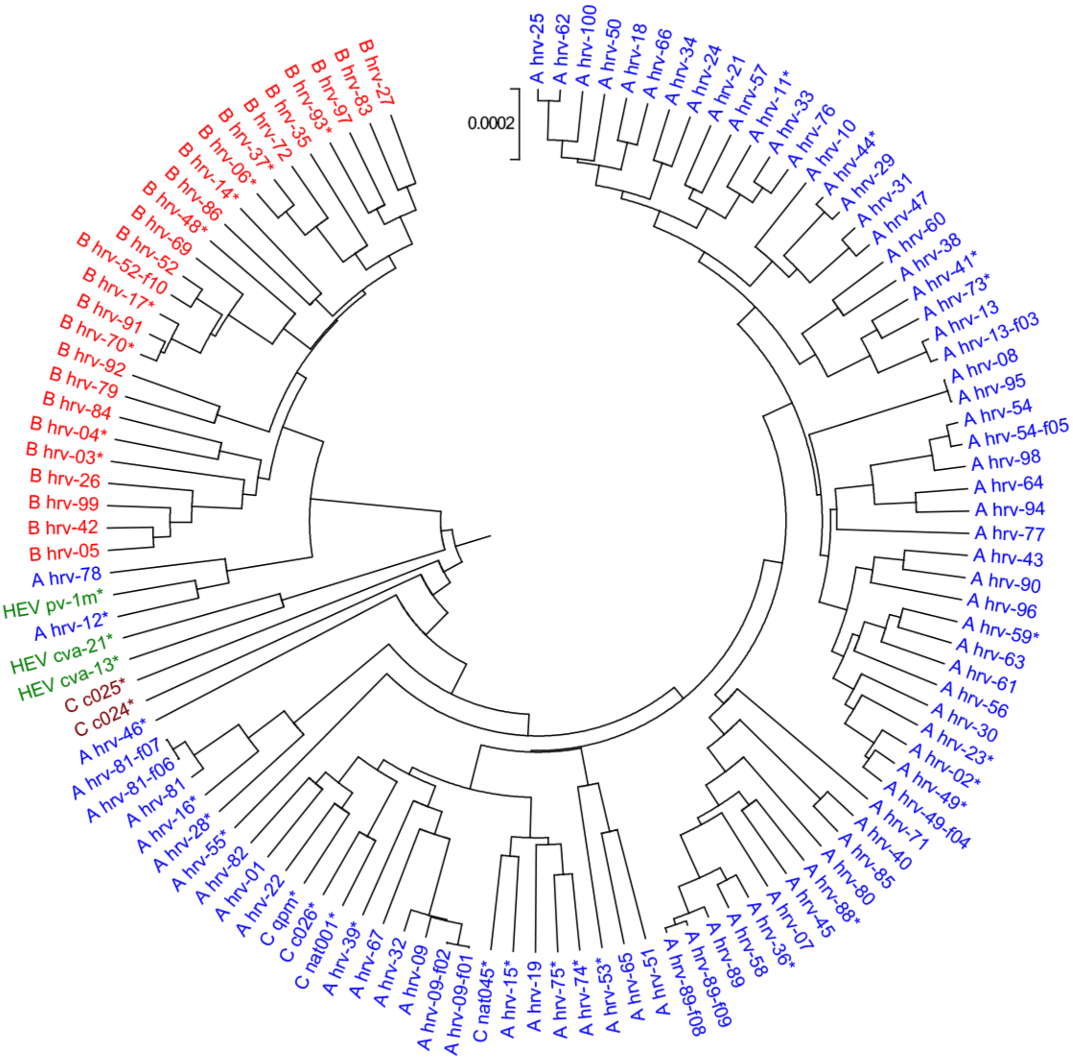

Figure S4. The UPGMA phylogenetic tree of 59 viruses in Ebolavirus genus based on feature frequency profiles method using 7 mer.

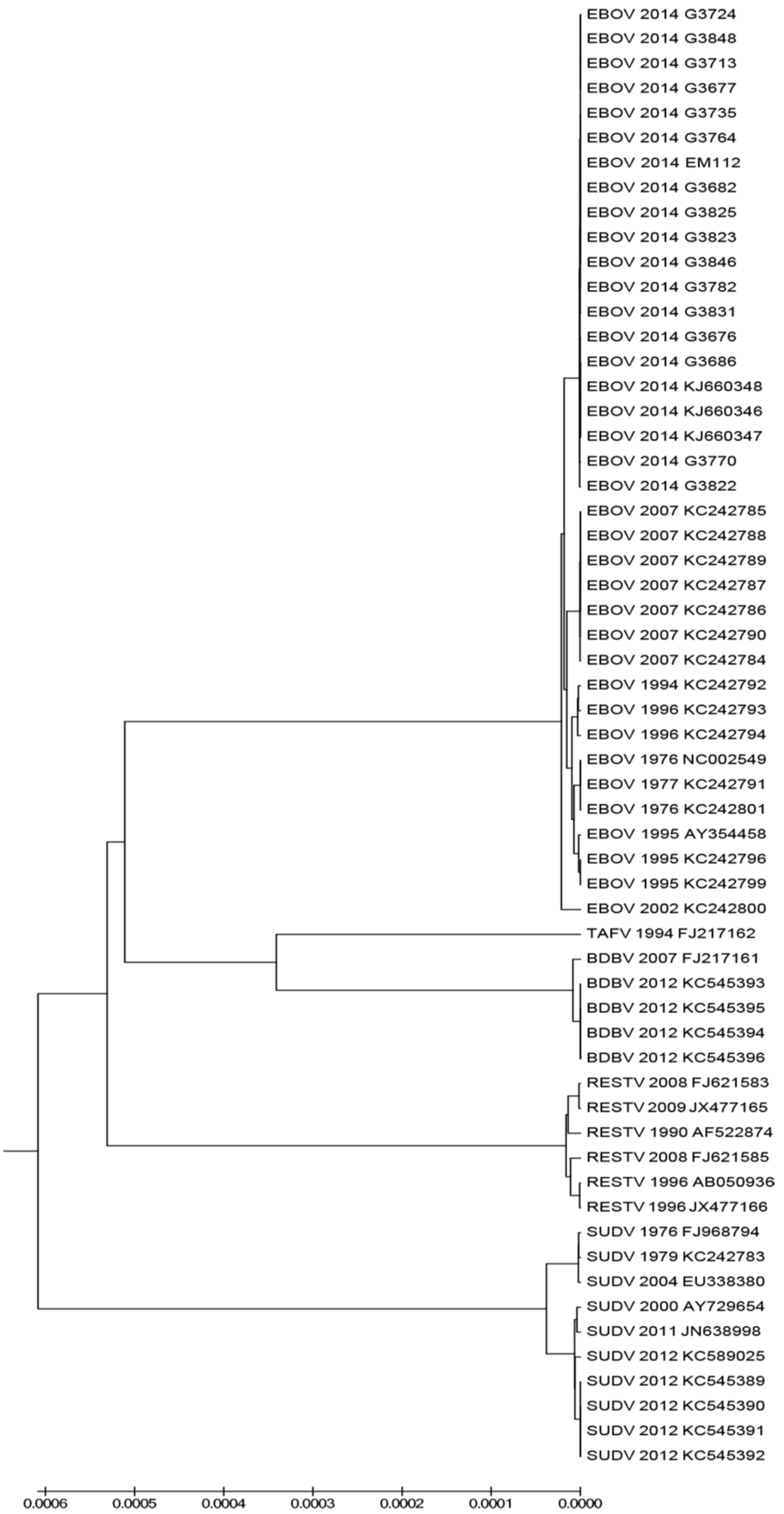

Figure S5. The UPGMA phylogenetic tree of coronaviruses based on feature frequency profiles method using 6 mer.

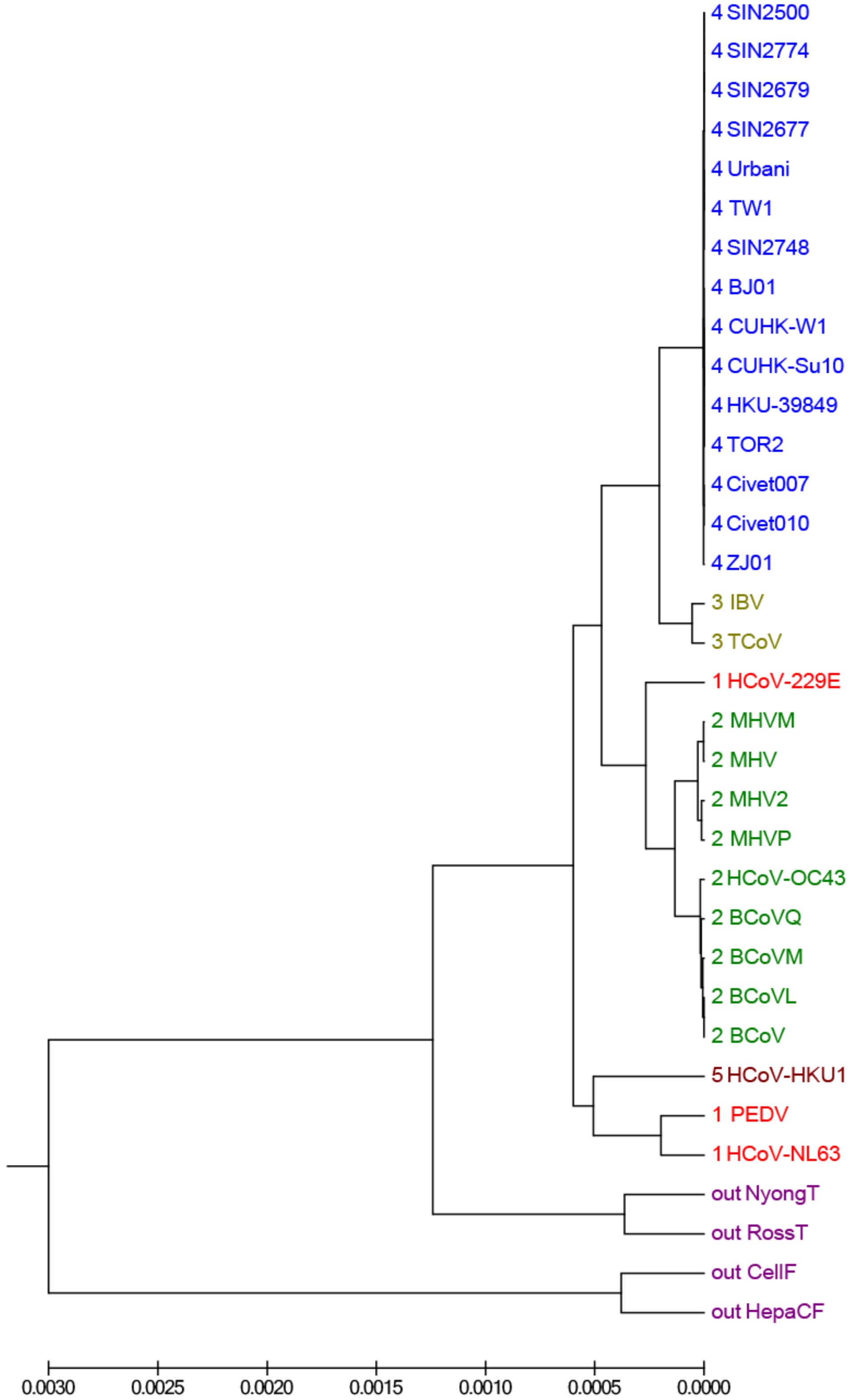

Figure S6. The UPGMA phylogenetic tree of 59 bacteria from 15 families based on feature frequency profiles method using 9-mer.

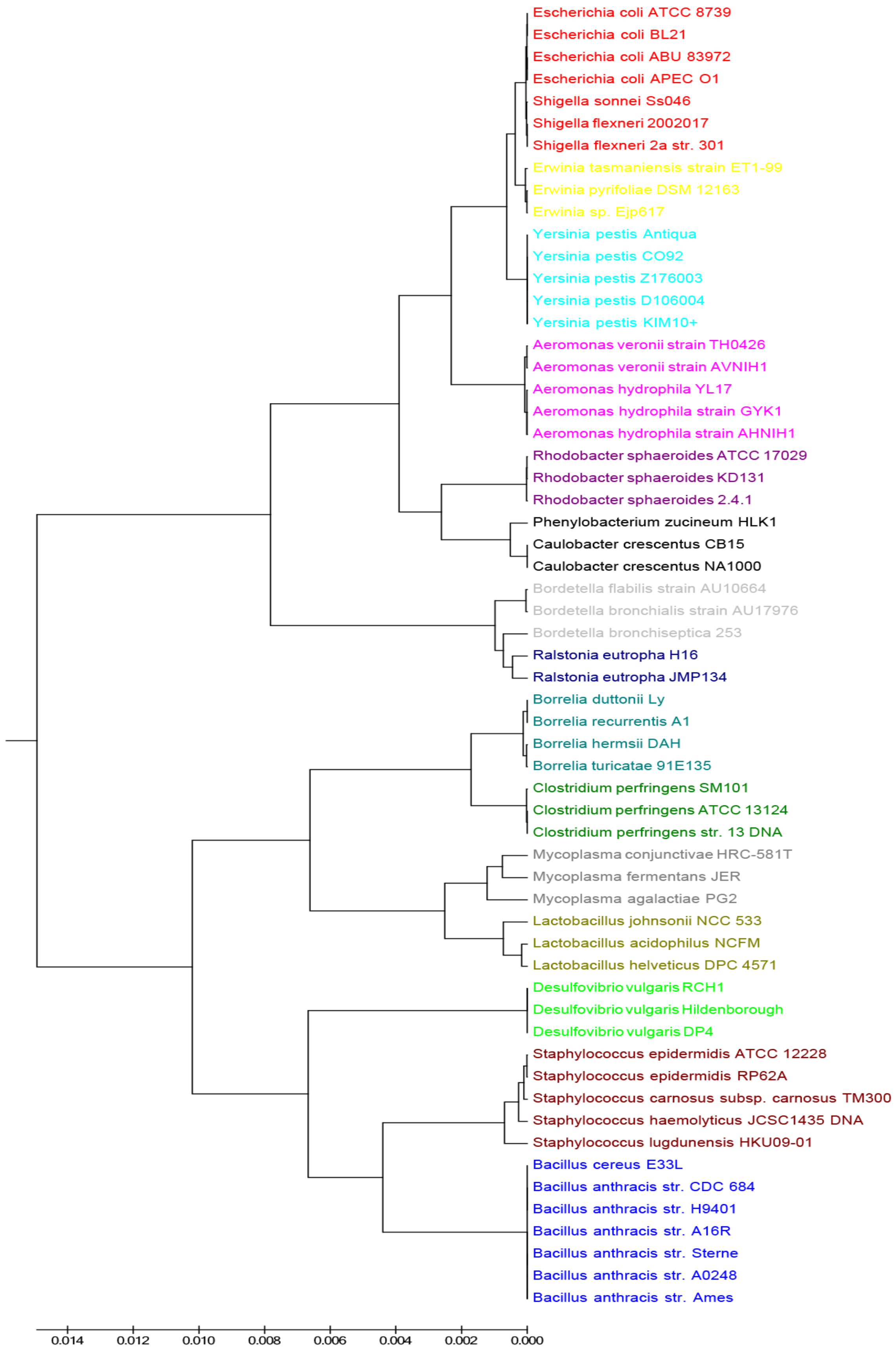

Figure S8. The UPGMA phylogenetic tree of 9 mammals based on our multiple encoding vector method.

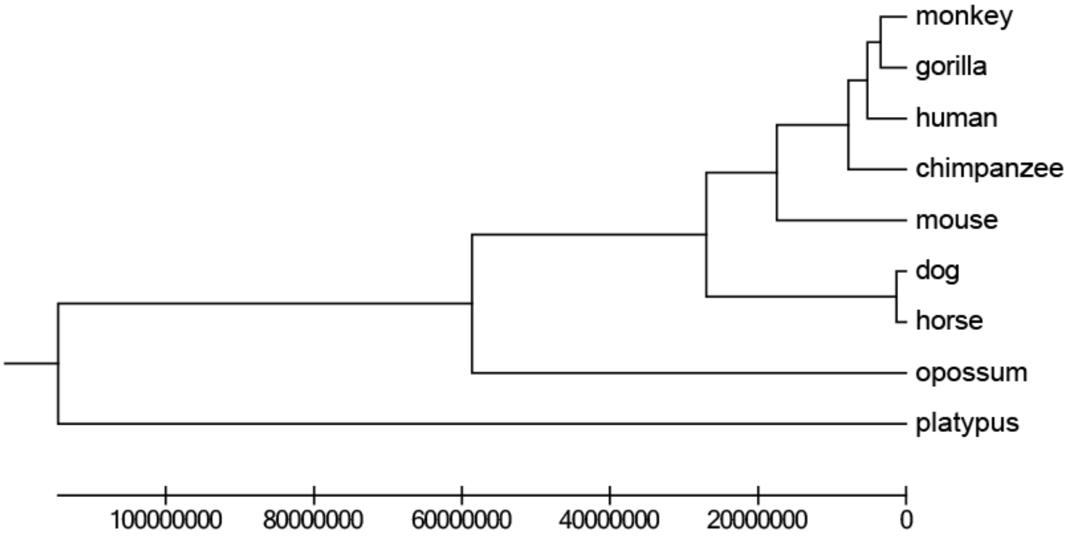

Phylogenetic trees with the ClustalW method

Figure S7. The UPGMA phylogenetic tree of 38 Influenza A viruses based on ClustalW.

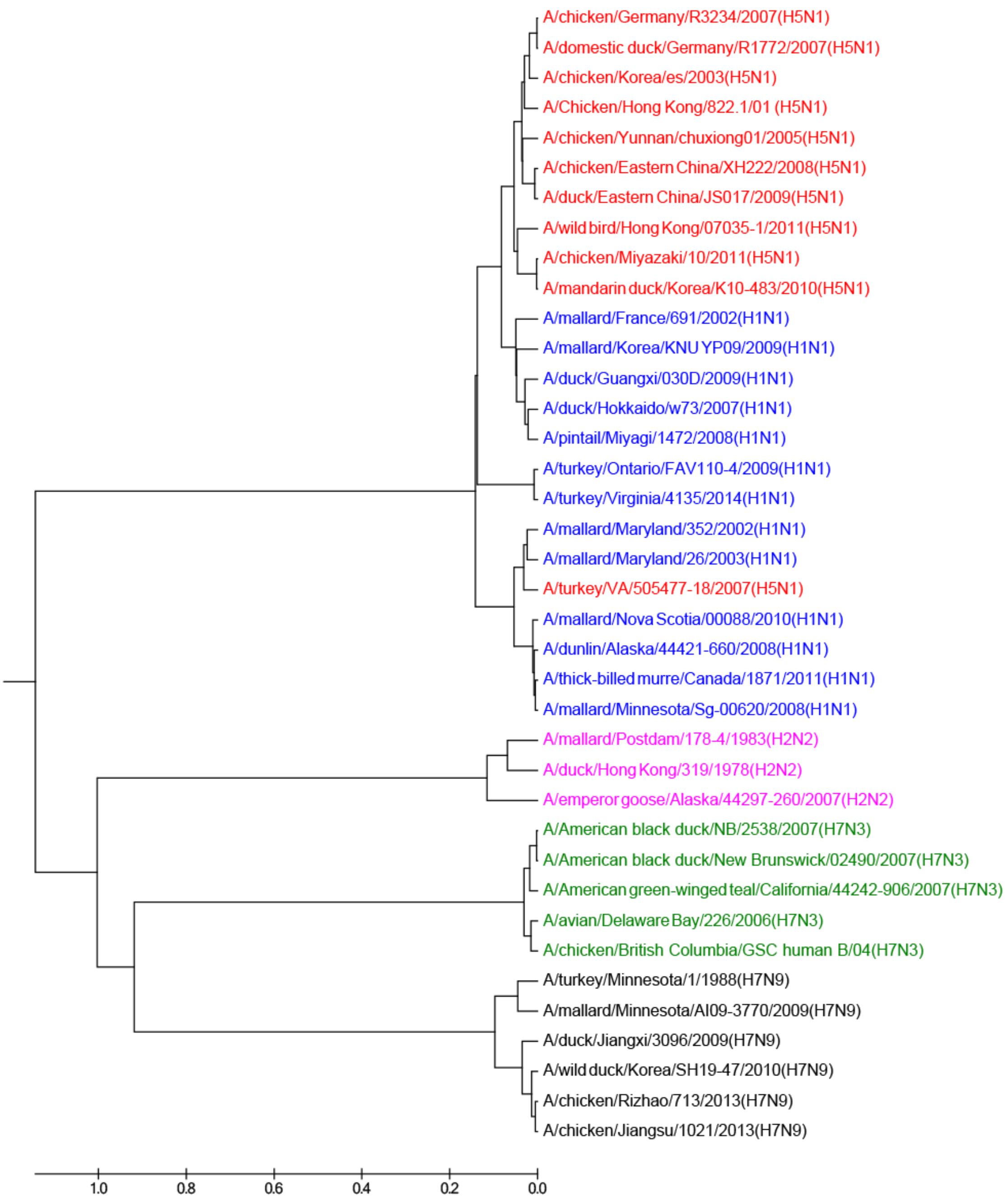

Figure M1. 41 mammalian mitochondrial genomes based on UPGMA algorithm with ClustalW

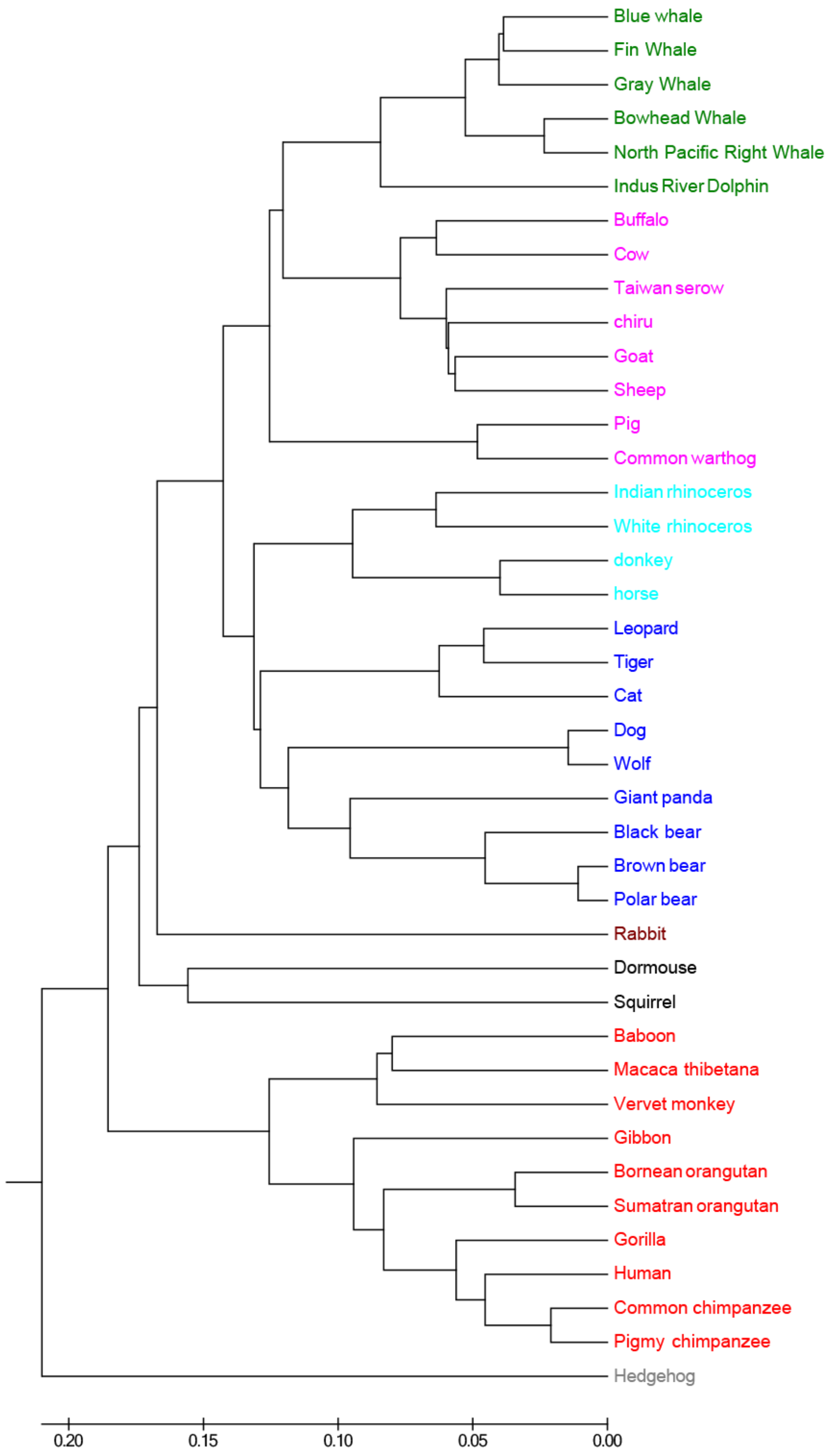

Figure M2. UPGMA tree of 38 Influenza A viruses by ClustalW

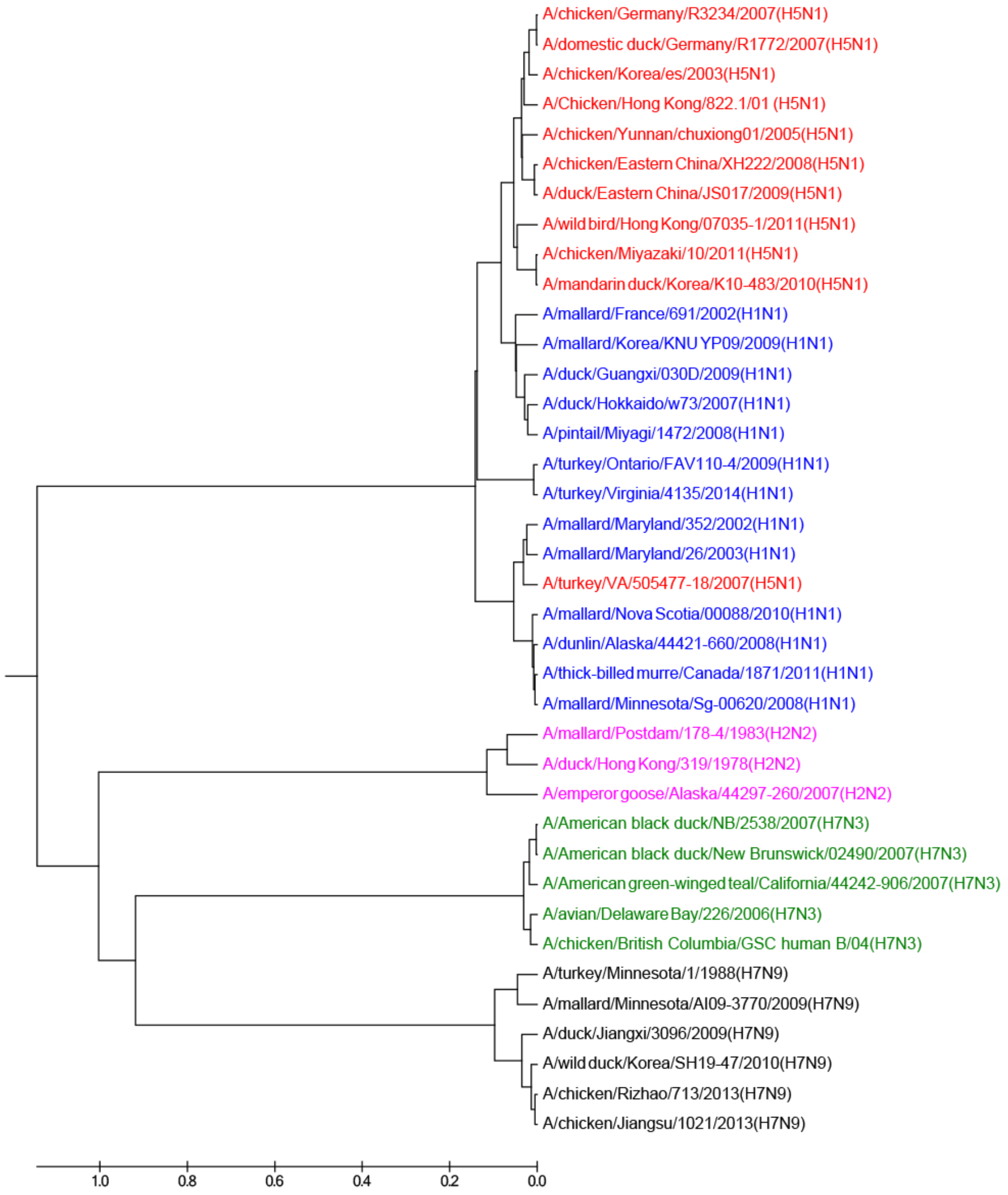

Figure M3. UPGMA phylogenetic tee of 113 HRV viruses and 3 outgroup by ClustalW

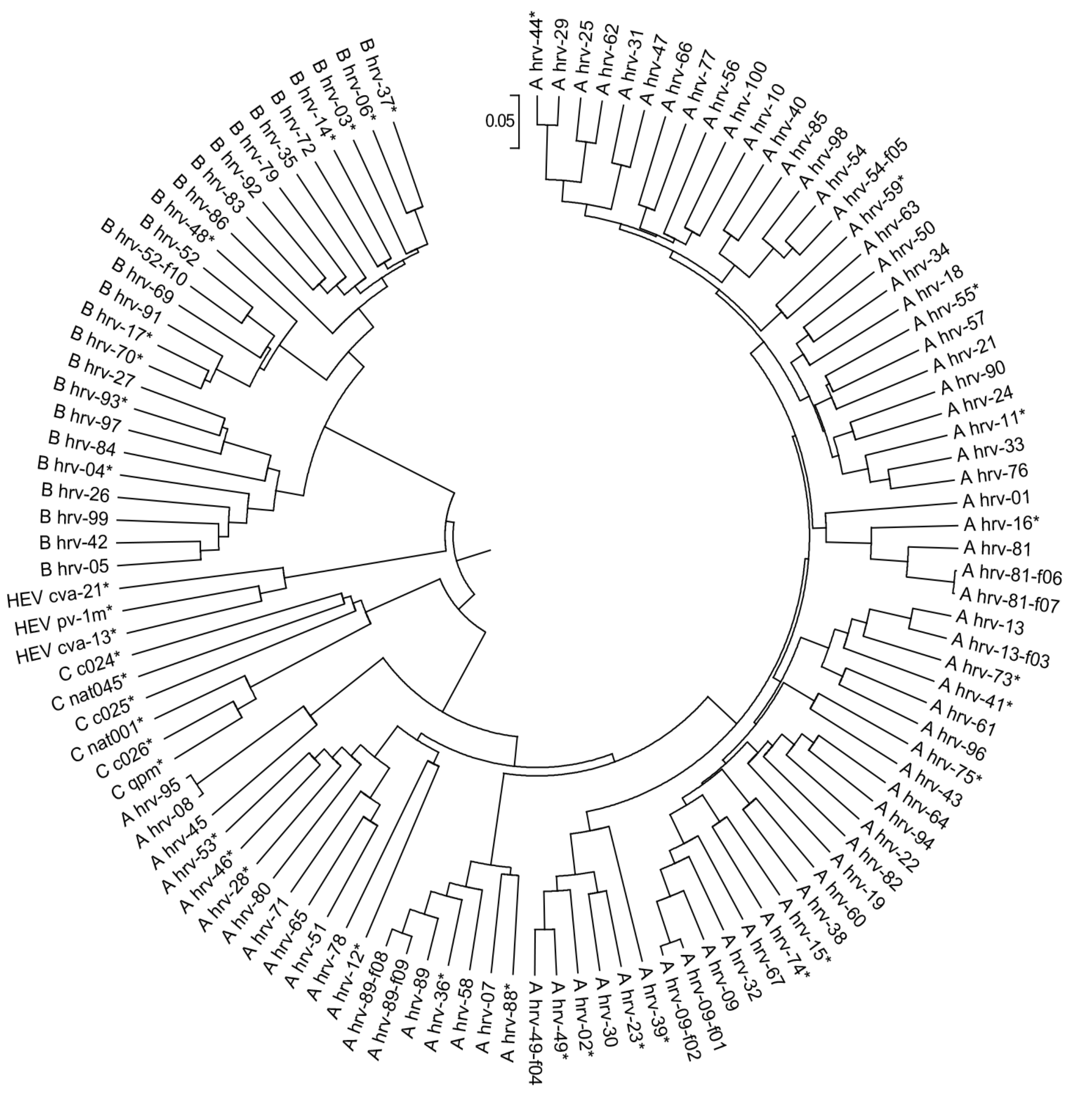

Figure M4. UPGMA phylogenetic tee of 59 ebolarviruses by ClustalW

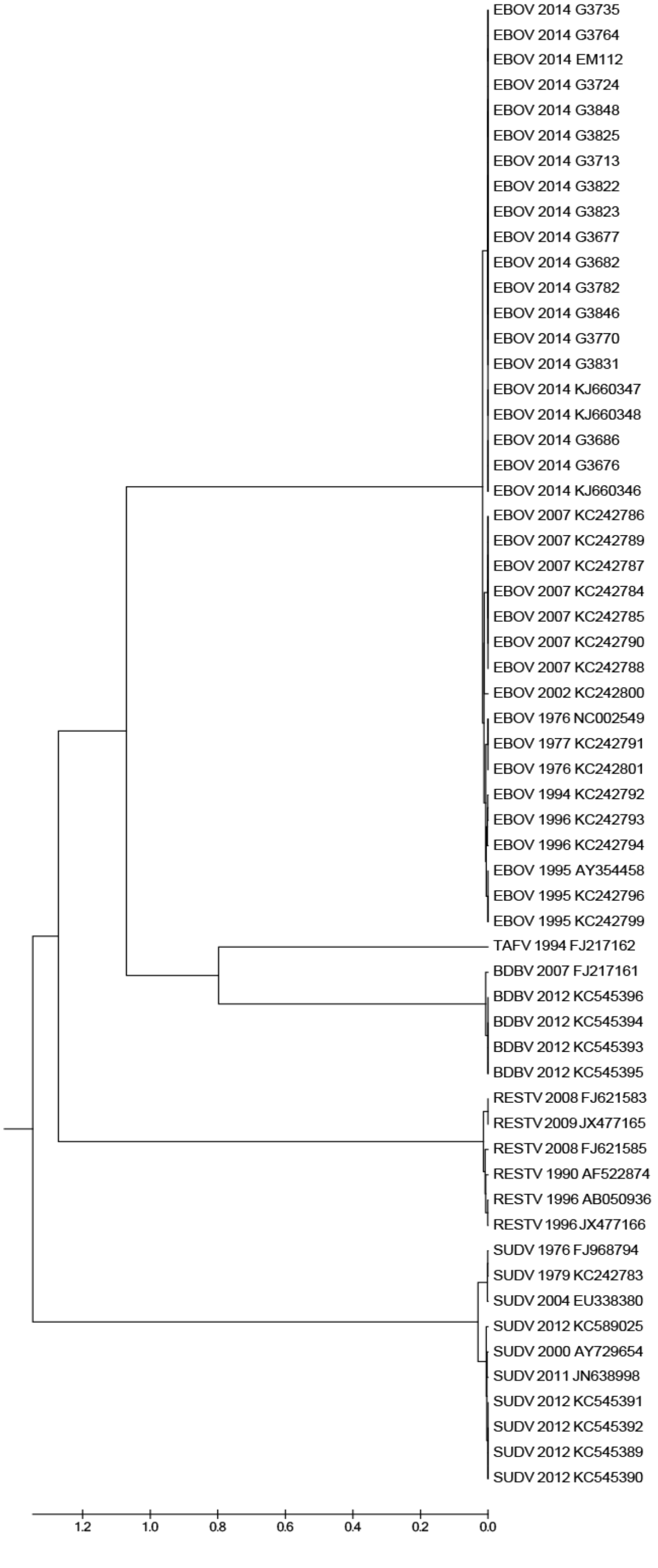

Figure M5. UPGMA phylogenetic tree of 30 coronaviruses and 4 extra non-coronaviruses by ClustalW

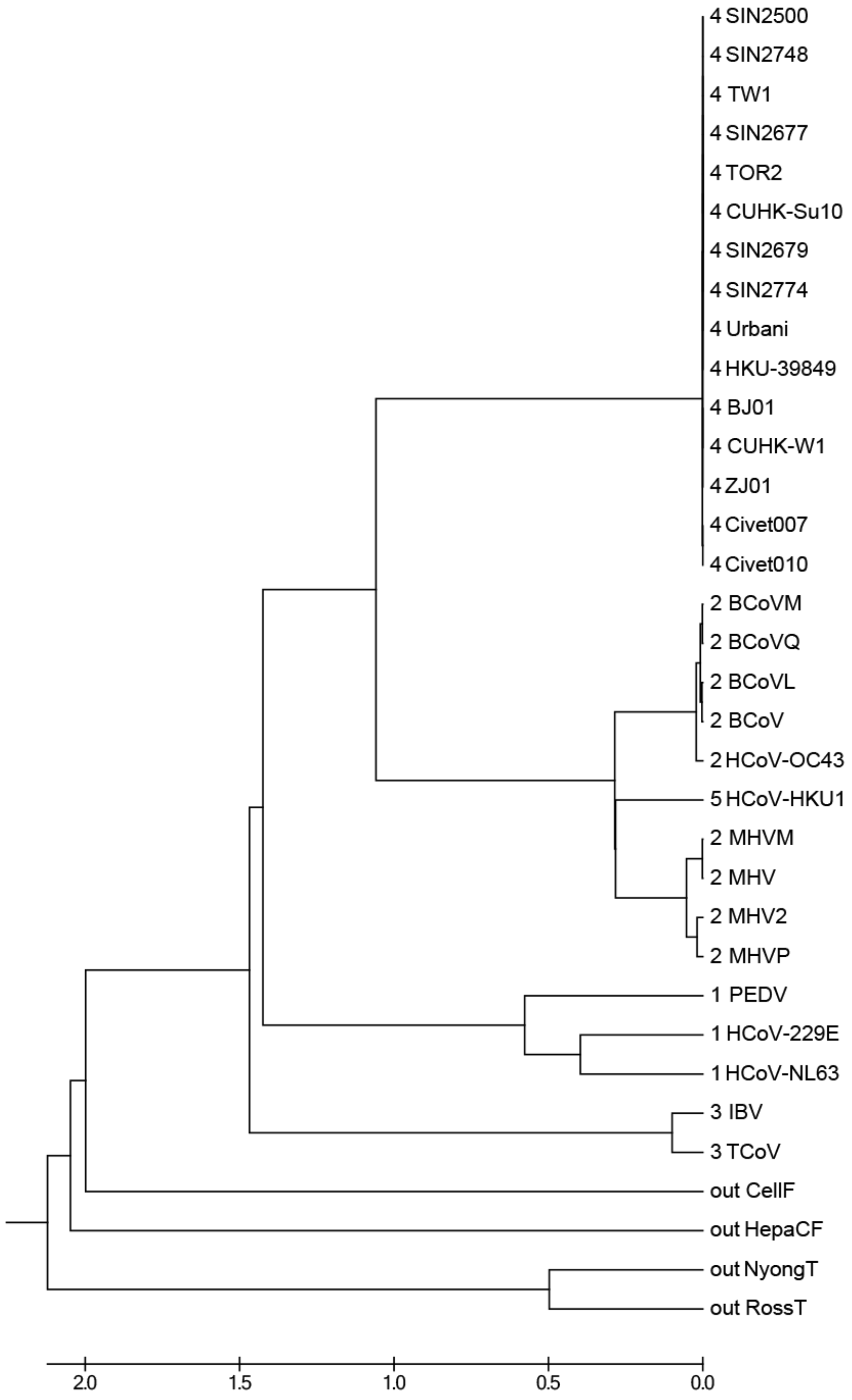

Supplement: Supplementary file 1 — Supplementary PDF File [file 41598_2017_12493_MOESM1_ESM.pdf]
